# The structural code of cyanobacterial genomes

**DOI:** 10.1093/nar/gku641

**Published:** 2014-07-23

**Authors:** Robert Lehmann, Rainer Machné, Hanspeter Herzel

**Affiliations:** 1 Institute for Theoretical Biology, Humboldt University, Berlin, Invalidenstraße 43, D-10115, Berlin, Germany; 2 Institute for Theoretical Chemistry, University of Vienna, Währinger Straße 17, A-1090, Vienna, Austria

## Abstract

A periodic bias in nucleotide frequency with a period of about 11 bp is characteristic for bacterial genomes. This signal is commonly interpreted to relate to the helical pitch of negatively supercoiled DNA. Functions in supercoiling-dependent RNA transcription or as a ‘structural code’ for DNA packaging have been suggested. Cyanobacterial genomes showed especially strong periodic signals and, on the other hand, DNA supercoiling and supercoiling-dependent transcription are highly dynamic and underlie circadian rhythms of these phototrophic bacteria. Focusing on this phylum and dinucleotides, we find that a minimal motif of AT-tracts (AT2) yields the strongest signal. Strong genome-wide periodicity is ancestral to a clade of unicellular and polyploid species but lost upon morphological transitions into two baeocyte-forming and a symbiotic species. The signal is intermediate in heterocystous species and weak in monoploid picocyanobacteria. A pronounced ‘structural code’ may support efficient nucleoid condensation and segregation in polyploid cells. The major source of the AT2 signal are protein-coding regions, where it is encoded preferentially in the first and third codon positions. The signal shows only few relations to supercoiling-dependent and diurnal RNA transcription in *Synechocystis* sp. PCC 6803. Strong and specific signals in two distinct transposons suggest roles in transposase transcription and transpososome formation.

## INTRODUCTION

Sequence periodicity, i.e. a regularly spaced bias in nucleotide frequencies along the DNA sequence, was reported for various genomic sequences since the 1980s (1,2). While in eukaryotes and archaea signals with period 10–10.5 bp are associated with the helical pitch of nucleosome-wrapped DNA (3,4), the causes and consequences of ∼11 bp period signals in bacterial genomes are less well understood (5–9). Dinucleotides usually yield a stronger signal than mononucleotides, and combinations of A and T (WW in IUPAC notation) often constitute the strongest signal (9), suggesting a mechanical interpretation: short runs of A and T nucleotides without the TpA step, a motif known as A-tract or AT-tract, induce a bend of the DNA backbone into the minor groove of the helix. If regularly spaced along the DNA polymer and in phase with the ∼10.5 bp pitch of the DNA double helix (‘phased AT-tracts’), this axial deformation can induce a persistent ‘intrinsic curvature’ of the DNA double helix (10). Differential periods of this phasing have been interpreted to correspond to underwinding or overwinding of the helix in negatively or positively supercoiled DNA (5,11) or to the two major conformations of negatively supercoiled DNA: plectonemically interwound DNA loops (period >10.5 bp) or solenoids (often denoted ‘toroidal’), wrapped around proteins such as the histone complex where the DNA helix itself is slightly overtwisted (period <10.5 bp) (12). Atomic force and electron microscopy experiments support the idea that helically phased AT-tracts preferentially lie in the loops of DNA plectonemes (13–16). Alternatively and in analogy to nucleosomes in eukaryotes and archaea, the signal might be related to the solenoidal wrapping around nucleoid-associated proteins, such as HU (17).

If residing in promotors or other regulatory sequences, sequence-directed DNA curvature can, e.g. position promoters at the apices of plectonemic DNA loops (18–21) where the torsional energy of negatively supercoiled DNA is locally channeled into unwinding of the double helix (22). Different dinucleotide periods (∼10.3 and ∼11 bp) in *Escherichia coli* promoters have been suggested to underlie differential transcription in response to changes of the extent of adenosine triphosphate (ATP)- and gyrase-dependent negative DNA supercoiling in bacteria (22–24).

Observed nucleotide periodicities in coding regions can also be induced by regularities in the amino acid sequence or RNA secondary structure. A 3 bp period signal can be partially attributed to codon usage bias (25–27), and this signal is potentially induced by RNA secondary structural code superimposed on the protein code (28,29). A specific pattern with 10–11 bp period and spanning only ∼30 bp is induced by the amino acid order of amphipathic α-helices (30,31), but this pattern can be readily distinguished from the ∼10 to 11 bp periodic signals of ∼100 bp length (5,9,32–33), which are preferentially encoded in the third codon position in both archaeal and bacterial (11,33) genomes. In *E. coli*, phased AT-tracts with period ∼11 bp and of length ∼100 bp were distributed in clusters along the genome, and covering both, intergenic and coding regions. Tolstorukov *et al.* suggested that this AT-tract distribution reflects a ‘structural code for DNA condensation into a nucleoid’ (32).

**Figure 1.**
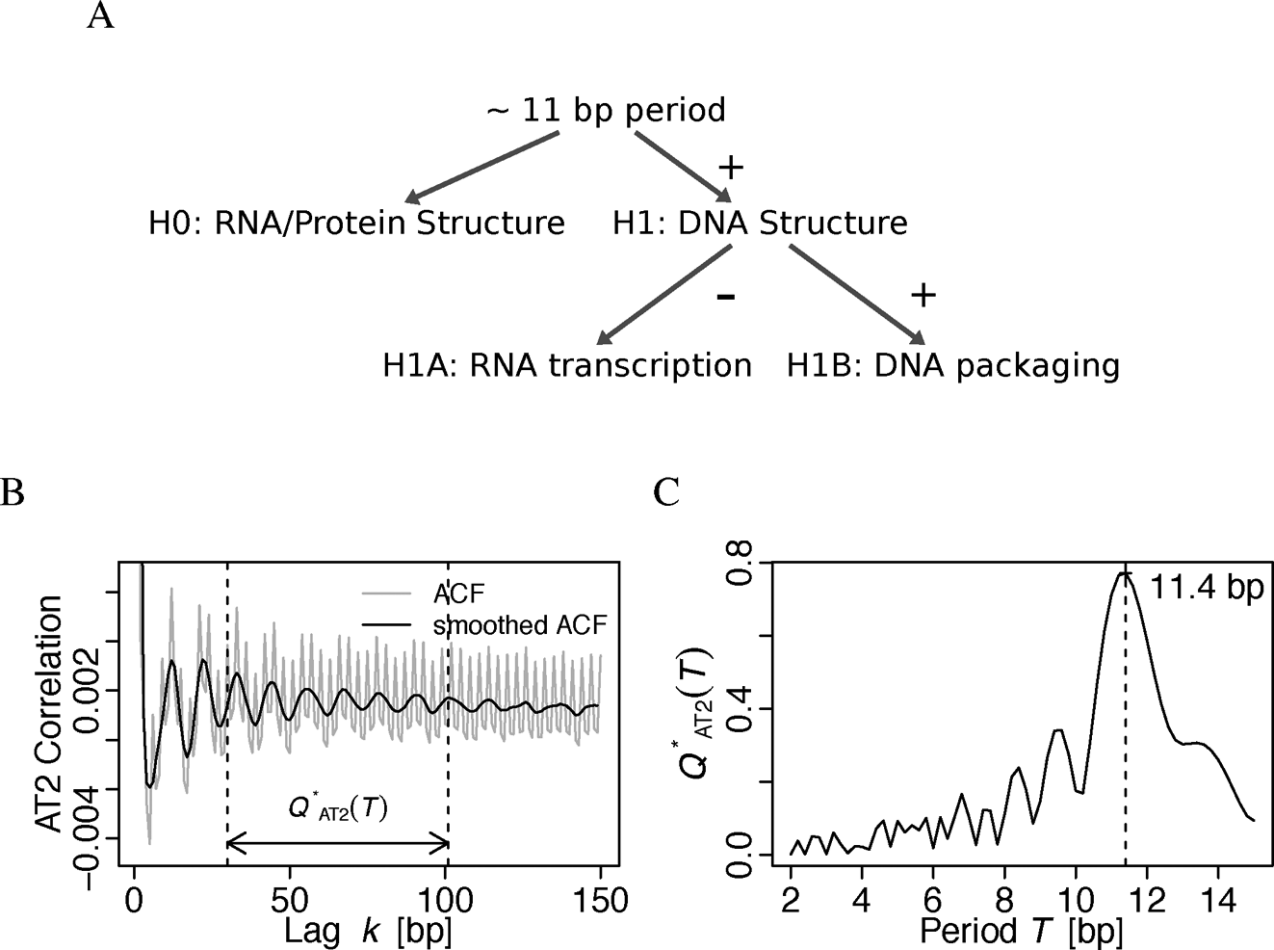
Approximately 11 bp dinucleotide periodicity in cyanobacterial genomes. (A) An *ad hoc* hypothesis tree on plausible interpretations of the ∼11 bp period in bacterial genomes, as discussed in the Introduction. Positive and negative evidence presented herein are indicated. (B) Autocorrelation function (ACF) of motif AT2 positions in the chromosome of *Synechocystis* sp. PCC 6803 before (*C*_*NN* − *NN*_(*k*), gray) and after smoothing by a window of 3 bp ($\bar{C}_{NN-NN}(k)$, black). (C) Power spectrum $Q^*_{AT2}(T)$ of the interval *k* = [30, 101] bp (indicated in the left panel) of the smoothed ACF and evaluated at periods *T* = [2, 15] bp. The dashed vertical line (right panel) indicates the maximum at 11.4 bp.

We summarize current hypotheses in an *ad hoc* inference tree (Figure 1A) and focus on the cyanobacterial phylum, phototrophic bacteria from which also plant chloroplasts descended. Cyanobacterial genomes had an especially strong signal at ∼11 bp in a previous comparative analysis (9). On the other hand, cyanobacterial chromosomes naturally oscillate between relaxed and negatively supercoiled states over diel (24 h) light/dark cycles (34). This oscillation is intimately involved in a genome-wide remodeling of the transcriptome (35). Thus, the suggested relations of the signal to negative DNA supercoiling (5), e.g. in supercoiling-dependent mRNA transcription (23,24) or DNA packaging (32), can be readily tested in a physiological context. Cyanobacteria are traditionally classified into 5 morphological subsections (36), that differ in the mode of cell division and include multicellular and differentiated organizations. However, several independent transitions in morphology are found throughout the cyanobacterial phylogeny (37–39). No ‘signature genes’ could be assigned to complex morphologies, but filamentous species tend to have a higher number of signaling and regulatory proteins (40).

In this first systematic overview of sequence periodicity in cyanobacteria, we find that a minimal motif of AT-tracts (WW without the TpA step) gives the strongest signal, supporting a general role in DNA structure. Loss of strong genome-wide periodicity is associated with transitions in cell morphology or lifestyle. A windowed scan allows to localize the signal along the genomes. The majority of the signal stems from protein-coding regions, where it is encoded preferentially in the first and third positions. We find no large-scale relation of the signal with supercoiling-dependent mRNA transcription, supporting a role in transcription-independent DNA transactions, such as DNA packaging. Finally, two distinct transposons show a strong ∼11 bp signal and we discuss potential functions in the formation of an active transpososome or regulation of transposase transcription.

## MATERIALS AND METHODS

### Genomes, lifestyle and phylogeny

Genomic sequences of 54 cyanobacterial strains were used, including genomes from a recent sequencing effort (40). For comparison we included two enterobacteriaceaeal species (*E. coli* K-12, *Dickeya dadantii* 3937), one archaeum (*Methanococcus maripaludis* S2) and one eukaryote (chromosome IV of *Saccharomyces cerevisiae*). Only sequences longer than 1 Mb were considered; plasmids and sequences annotated as unfinished were excluded. All genomic sequences and genome annotations, protein-coding (CDS) and intergenic (non-annotated sequences) segments, were obtained from NCBI (National Center for Biotechnology Information) GenBank (41) or the JGI (Joint Genome Institute) database (42). Phylogenetic trees were obtained from the authors of ref. (40), based on 31 conserved proteins, and from the IMG (Integrated Microbial Genomes) database (43), based on 16S rRNA alignments of the SILVA database (44). Species lifestyle information were obtained from the IMG database and the supplemental material of ref. (40). Biological function annotations and InterProScan protein domain matches for CDS of the genomes of *Synechocystis* sp. PCC 6803 and *Cyanothece* sp. 8801 were obtained from the CyanoBase database (45). A table of the used sequences, their sources and results reported herein is provided as Supporting File S1 and described in the Supporting Material PDF.

### Transcriptome data

The diurnal time-series data from *Synechocystis* sp. PCC 6803 (46) (GEO: GSE45667) was processed and clustered into co-transcribed gene cohorts as described previously (46,47). In short, the Discrete Fourier Transform (DFT) was calculated from raw microarray fluorescence data of 3370 protein-coding transcripts, where time-series were concatenated from biological duplicates, each measured over one 24-h light/dark cycle. The highest frequency component was removed, accounting for measurement noise without data normalization. The remaining 5 DFT components where then clustered by the model-based clustering tool flowClust (48), choosing cluster number *k* = 10 and assigning all genes annotated but absent from the microarray to cluster 11. The supercoiling-sensitive gene cohorts were taken without further processing from the supplementary material of (49), where group 1 are genes that were consistently ‘up’-regulated by increased gyrase-mediated negative supercoiling in a series of experiments, group 3 genes were consistently ‘down’-regulated, group 2 genes showed a ‘mixed’ response and ‘nr’ were non-responsive genes. A table of all analyzed CDS data is provided as Supporting File S2 and described in the Supporting Material PDF.

### Dinucleotide periodicity measures and statistics

To assess both genome-wide and local (windowed) dinucleotide motif periodicities, we use the autocorrelation function (ACF) as detailed in (8) with a window smoothing of width 3 bp to suppress the strong contribution from coding regions. A normalized power spectrum $Q^*_{NN}(T)$ of the ACF is then calculated after (9) *via* the Fourier transform of the ACF between *k*_min_ = 30 bp and *k*_max_ ≈ 101 bp, i.e. excluding shorter-range signals (<30 bp) induced by amphipathic α-helices (5). Figures 1B and C exemplify this procedure and a comprehensive account of our approach is provided in the Supporting Methods.

### Windowed periodicity analysis

To exclude effects of sequence composition we randomly permuted the sequence of each window (200 bp) *i* using uShuffle (50) with preservation of dinucleotide content, and recalculated its Fourier spectrum. Where indicated (200Avg4), spectra from four consecutive sequence windows were averaged. A *P*-value *P*_*T*, *i*_ for each spectral component (period) *T* was then calculated from 5000 permutations and used to select significantly periodic windows. See the Supporting Methods for motivation and details.

### Overlaps of periodic windows with annotated features

Adjacent significantly periodic windows (*P*_*T*, *i*_ < 0.01) within four period ranges were concatenated to yield four distinct sets of non-overlapping periodic genome segments. The significance of the overlap between these segments and protein-coding (CDS) and intergenic segments was tested using the Jaccard test with interval permutation as implemented in the R package GenometriCorr (51).

### Codon permutations

A customized version of the R package seqinr was used to perform codon order permutation, synonymous codon replacement (without any codon usage bias) and individual codon position permutations (with preservation of the original base composition). The effects of permutations were quantified by calculating the ratios of the signal $Q^{CDS}_{AT2}(T)$ at *T* = 11.8 bp in concatenated CDS before and after permutation (Supporting Methods).

### CDS cluster enrichments

CDS periodicity clusters were tested against a variety of gene-level annotations and data sets using cumulative hypergeometric distribution tests. We consider *P*-values from these scans as a measure of enrichment and therefore do not control for false discovery rates. The main reported observations remain significant also when corrected by the Benjamini–Hochberg method (all *P*-values are listed in Supplementary Tables S1 and S2).

## RESULTS

### Sequence periodicity across the cyanobacterial phylum

We focussed on periodic enrichment of dinucleotide motifs (*NN*) as a minimal unit of DNA structure-related sequence context (52,53). The motifs WW (all combintations of A and T) and AT2 (WW without TpA: a minimal motif of AT-tracts) were included due to prior results on bacterial genome periodicity (5,8,9). Their genome-wide periodicity strengths were quantified as a signal-to-noise ratio $Q^*_{SNR}(NN)$ for the period range 10–12 bp (Figure 1 and Supporting Methods).

**Figure 2.**
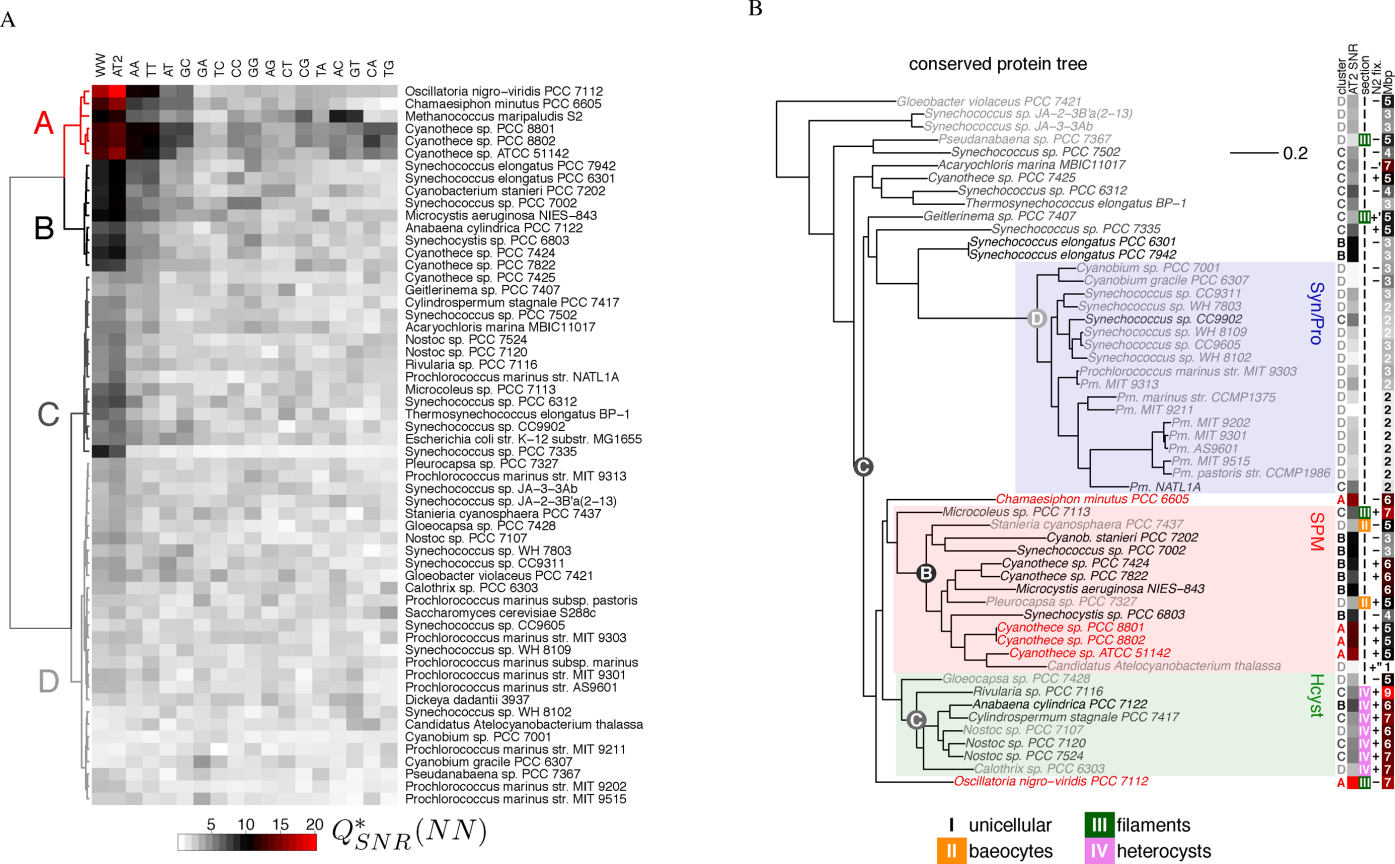
Periodic dinucleotides across the cyanobacterial clade. (A) The power spectrum signal-to-noise ratio $Q^*_{SNR}$ for periods 10–12 bp (color-coded) for all possible dinucleotides NN, WW and the AT-tract motif AT2 (columns) and for genomes of 54 cyanobacterial and 4 ‘control’ species (rows). Hierarchical linkage clustering was performed using Ward's method for species (tree on the left) and ‘complete linkage’ for dinucleotides, and cut at level *k* = 4 to obtain clusters A–D. (B) The phylogenetic tree was obtained from the authors of (40) and is based on alignments of 31 conserved proteins. The colored clades were bootstrap-supported at ≥70%. The columns on the right show (in order): the clustering of species and their $Q^*_{SNR}(AT2)$ from Figure 2A; the morphological sections (I–IV); whether they are able to fix nitrogen; and the genome length in Mbp (without plasmids). Ancestral states (cluster assignments and color-coded $Q^*_{SNR}(AT2)$ at internal nodes) were inferred using maximum-likelihood methods (Supporting Methods). All species data are provided in Supporting File S1.

Data exploration by clustering and principal component analysis of the $Q^*_{SNR}(NN)$ profiles of 54 cyanobacterial and 4 reference genomes confirm that WW dinucleotide combinations carry the strongest signal (Figure 2A and Supplementary Figure S1). Of all WW combinations, the TpA dinucleotide has the lowest contribution to the main component PC1 (Supplementary Figure S1A), consistent with the structurally distinct properties of this dinucleotide step (10). Consequently, the AT2 motif gives the strongest signal in 35 species, followed by WW in eight species. Our coarse clustering of species (clusters A–D in Figure 2A) reflects mainly the strength of the AT2 signal. Only the three highly periodic *Cyanothece* strains carry additional periodicity in CpA and TpG dinucleotides (cluster A in Figure 2A, PC3 in Supplementary Figure S1B). Only the archaeum *Methanococcus marsipaludis* S2 features additional periodicities in ApC and GpT dinucleotides (Figure 2A, PC2 in Supplementary Figure S1A).

AT2 spectra of all 58 genomes confirm that almost all cyanobacteria harbor a genome-wide AT2 signal at the typical period of 11–11.6 bp (Supplementary Figures S2– S6). The two enterobacteriaceaeal reference species (maximum at ∼11bp) and budding yeast (9.8 bp) show a comparatively weak and the archaeum (9.8 bp) a very strong signal.

#### Phylogeny and lifestyle

Strong genome-wide AT2 periodicity is only conserved in SPM (*Synechocystis, Pleurocapsas, Microcystis*, (38)), one of three well supported cyanobacterial clades (Figure 2B and Supplementary FigureS7A) (37–40). It consists mostly of unicellular bacteria which reproduce by binary fission or budding (morphological section I, (36)). All four weakly periodic strains of this clade underwent transitions in cellular morphology or lifestyle: two strains are baeocyte forming cells from section II, which are characterized by multiple fission into several small (greek baeo-) daughter cells (54); and *Candidatus Atelocyanobacterium thalassa* (also known as cyanobacterium UCYN-A) lives as a nitrogen-providing symbiont of a unicellular alga and has strongly reduced genome and metabolic capacities (55). At the base of the SPM clade is the only filamentous species (section III), and its intermediate level of AT2 periodicity is consistent with most other filamentous species including the heterocyst-forming filaments (section IV) in clade Hcyst (heterocysts are differentiated cells specialized in nitrogen-fixation). Two highly periodic strains from sections III and I are found at the base of sister clades Hcyst/SPM. Their position had little bootstrap support and they branch within Hcyst in a 16S rRNA-based tree (40) (Supplementary Figure S7A).

In contrast, the Syn/Pro clade (picocyanobacteria of section I) has very weak genome periodicity. This clade is characterized by very small cells and stream-lined genomes encoding for a minimal oxyphototroph lifestyle (56), with a strict coupling of S-phase to cell division, i.e. a monoploid lifestyle (57,58). The lack of periodicity in this clade underlies several significant (anti-)correlations of the signal with general properties such as cell size, genome length or the fraction of metabolic genes (Supplementary Figure S7B). Bootstrap support for the position of *Synechococcus elongatus* species, which have a strong genome periodicity in cluster B, was low (40) and they may instead also branch basal to the SPM and Hcyst clades (38). Distantly related and underrepresented species outside of the three main clades all have intermediate or weak genome periodicity, thus it is possible that a high genome periodicity has evolved within the SPM/Hcyst sister clades. The two highly periodic species at their base make it difficult to infer the periodicity strength of their common ancestor, i.e. whether genome-wide AT2 periodicity at ∼11 bp was gained by SPM or lost by Hcyst.

In summary, the minimal AT-tract motif AT2 shows the most pronounced genome-wide periodicity, consistent with previous interpretations of a role of phased AT-tracts in supercoiling-dependent DNA packaging mechanisms (32). High genome-wide periodicity is mainly found in the unicellular SPM clade of cyanobacteria, and loss of periodicity accompanies morphological or lifestyle transitions.

### AT2 periodicity and the protein code

To track the genomic locations of strong genome-wide AT2 periodicity, we calculated the normalized Fourier spectra $Q_{AT2}^*(T)$ for 200 bp windows along the genomes of 10 representative species, from strong to weak genome-wide periodicity. Each window is assigned to a period *T*_sig_, which achieves the smallest *P*-value in permutation tests (Supporting Methods). In agreement with a previous windowed scan (9), the distribution of windows with period *T*_sig_ (Supplementary Figure S8A–J) reveals an additional peak at ∼10 bp in four *Cyanothece* sp. and *Synechocystis* sp. PCC 6803. An additional averaging of AT2 spectra $Q_{AT2}^*(T)$ over four adjacent 200 bp windows (200Avg4) reduces the signal-to-noise ratio and emphasizes the very faint ∼11 bp peaks in weakly periodic species, e.g. in *E. coli* , but decreases the resolution between the bimodal peaks at ∼10 and ∼11 bp.

**Figure 3.**
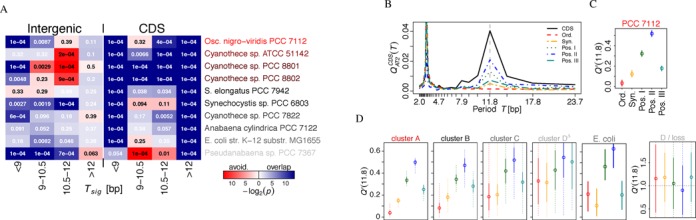
AT2 periodicity and the protein code. (A) The overlap of periodic genome segments with coding sequences (CDS) and with intergenic regions was investigated by Jaccard tests (51). Genome segments were concatenated from adjacent significantly periodic windows of 200 bp length in four period ranges (columns). Species name colors indicate their $Q^*_{SNR}(AT2)$ as in Figure 2. (B) The $Q^{CDS}_{AT2}(T)$ of codon-permuted and concatenated protein-coding regions (CDS) of *Oscillatoria nigro-viridis* PCC 7112 for the original coding sequences (CDS), and the mean spectra of 50 permutations: codon order permutation (Ord.), synonymous codon replacement (Syn.) and permutations of only the first (Pos. I), second (Pos. II) or third (Pos. III) codon positions. (C) The fraction *Q*′(*T*) of the unpermuted signal at *T* = 11.8 bp (vertical line in B) remaining after permutations (open circles are the means and vertical lines indicates the range of sampled values in 50 permutations). (D) As Figure 3C but summarized for the species clusters from Figure 2A (without non-cyanobacteria), where ‘D$^\Delta$’ is without the four species in ‘D/loss’ (solid lines indicate the standard deviation, dashed lines the full range) and for *E. coli* str. K–12 substr. MG1655. The full spectra of representative species for all 58 species are shown in Supplementary Figures S2–S6.

Next, we concatenated adjacent significantly periodic windows, pooled by ranges of periods. The resulting non-overlapping periodic genome segments were tested for overlaps with annotated protein-coding (CDS) or intergenic sequences (Figure 3A). Segments with periods of ∼11 bp significantly overlap with coding regions in most species, and in *Cyanothece* sp. even significantly avoid intergenic regions. In contrast, especially windows at lower periods of ∼10 bp tend to overlap with intergenic regions in species with weaker genome-wide signals and in the highly periodic *Oscillatoria nigro-viridis* PCC 7112. These intergenic enrichments are consistent with many previous observations on curvature in bacterial promotor (18–21,23,24) and transcription termination (59) regions. But the increased genome-wide signal in highly periodic cyanobacteria cannot be explained by promoter curvature and stems mostly from periodicity embedded into protein-coding regions.

#### Codon positions

It was previously established for archaeal, bacterial and eukaryotic genomes, that nucleotide periodicities can overlap with the triplet code and are found most pronounced in the synonymous third codon position (11,33). To test such an integration with the protein code, we calculated the ACF spectra $Q^{CDS}_{AT2}(T)$ for discrete Fourier components of all concatenated protein coding regions (CDS) before and after different codon shuffling and permutation strategies. The effect on the ∼11 bp signal is summarized by *Q*′(*T*), the fraction of the original signal probed at *T* = 11.8 bp that remains after permutation (Figure 3B–D, Supplementary Figures S2–S6).

Shuffling the codon order, which destroys the amino acid sequence but preserves the codon usage bias, removes the ∼11 bp period completely, i.e. *Q*′ is between 0% and 10% in species with a strong genome-wide signal (Figure 3D, Supplementary Figures S2 and S3). To test whether secondary effects of amino acid sequences, such as amphipathic α-helices or other regularities, may induce the signal, we performed synonymous codon replacements which maintains the original amino acid sequence but changes codon usage. This permutation still reduces the signal to ca. 10–20%, confirming that the dinucleotide signal is encoded predominantly at the synonymous position III. Selective permutations of positions I and III severely decrease the ∼11 bp amplitude to 20–40%, with a stronger effect of position III, while permutation of position II yields the lowest signal reduction (highest *Q*′) to ca. 50% of the original level. The same but less pronounced trends are observed in most species with a weaker genome-wide signal and including all non-cyanobacterial species (Figure 3D, Supplementary Figures S4 and S5). Expectedly, the archaeum and yeast peak at ∼10 bp, while *E. coli* peaks at both periods. A complete loss of the signal can only be observed in three picocyanobacteria, where permutations have on average no effect; and in the symbiont UCYN-A, where they even enhance the ∼11bp signal (Figure 3D and Supplementary Figure S6).

### Diurnal and supercoiling-sensitive transcripts

**Figure 4.**
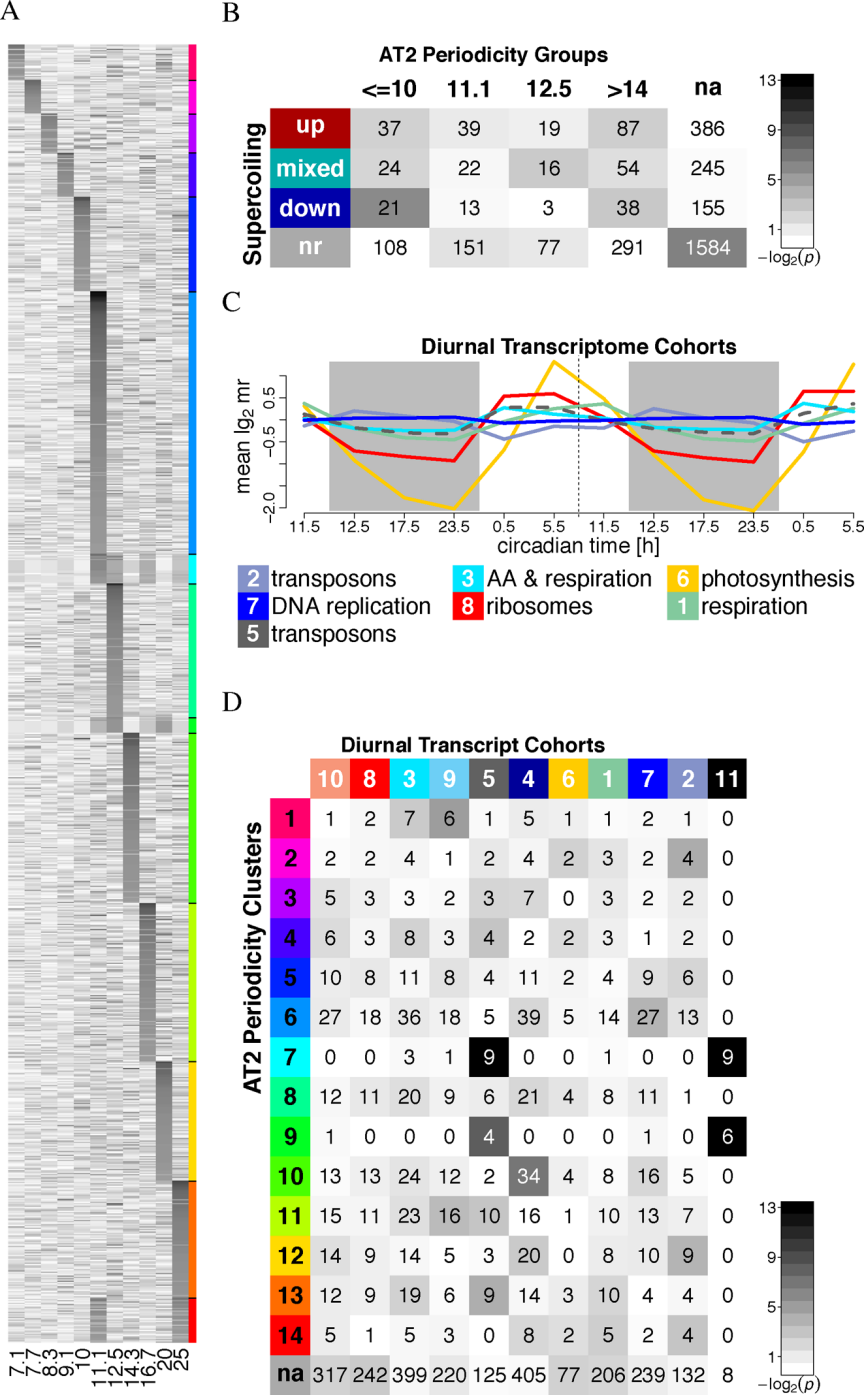
Periodicity in PCC 6803 coding regions. (A) 1000 coding sequences of *Synechocystis* sp. PCC 6803, clustered by their AT2 periodicity spectra $Q^*_{AT2}(T,i)$ (color-coded, with black indicating higher values). The period *T* (in bp) is shown on the *x*-axis for all coding sequences *i* on the *y*-axis. The cluster membership (1–14 from top to bottom) of coding sequences is shown color-coded on the right. (B) Cluster overlap profile. CDS periodicity clusters were comprehended into groups with similar main period *T*_max_ (columns, see table in Supplementary Figure S10A) and analyzed for overlaps with genes ‘up’-regulated, ‘down’-regulated and genes that showed a ‘mixed’ or no (nr) response to experimentally manipulated levels of DNA superpcoiling (rows, from (49)). The numbers are the genes shared by the respective clusters and the color code indicates the *P*-values derived from cumulative hypergeometric distribution tests for enrichment and without correction for multiple testing to show unbiased and comparable overlap profiles. (C) Mean transcript abundance time-series of diurnally co-transcribed cohorts. Only cohorts that also show typical features of supercoiling-sensitivity (function, strong bias in GC-content, Supplementary Figures S11 and S12), are shown. (D) Cluster overlap profile of diurnally co-transcribed ((46), Supplementary Figure S11) gene cohorts (columns) with the CDS periodicity clusters (rows). All transcriptome-based and the CDS periodicity clusters are provided in Supporting File S2.

Next, we clustered the 1000 most periodic CDS of *Synechocystis* sp. PCC 6803 and *Cyanothece* sp. PCC 8801 by their spectra $Q^*_{AT2}(T)$, yielding clear subgroups of genes which share a main period and similar spectra (Figure 4A, Supplementary Figures S9 and S10, Supplementary Tables S1 and S2). CDS with the main periods *T*_max_ at 10–12.5 bp constitute the largest groups, especially in PCC 8801 and consistent with its higher genome-wide periodicity.

Curved DNA and phased AT-tracts downstream of the promoter could also affect transcription by plectonemic DNA looping (19,60,61). Thus, we compared the CDS periodicity clusters with two transcriptome data sets that reflect supercoiling-sensitive transcription in *Synechocystis* sp. PCC 6803 in both experimental intervention (49) and in physiological (diurnal) context (46). Only when collapsing our CDS periodicity clusters by ranges of their main periods *T*_max_, we find two statistically weak overlaps with the transcriptome groups from ref. (49) (Figure 4B): genes that were non-responsive to experimental changes in DNA supercoiling (49) are enriched in un-clustered (weakly or a-periodic) genes (*P* = 0.012), and genes down-regulated by increased supercoiling are slightly enriched with genes with *T*_max_ ≤ 10 bp (*P* = 0.017).

We recently reported a diurnal transcriptome study from *Synechocystis* sp. PCC 6803 and clustered all transcript time series into co-transcribed gene cohorts defining a functionally coherent gene expression program (46) (Supplementary Figure S11). In contrast to CDS periodicity clusters, the transcript cohorts of this time-series show highly significant overlaps with supercoiling-sensitive transcript groups (Figure 4C and Supplementary Figure S12). They show the typical strong bias in GC-content (Supplementary Figure S12B) that has been reported from supercoiling-sensitive genes in *E. coli* (62) and *S. elongatus* PCC 7942 (35): GC-rich genes that encode for amino-acid synthesis and ribosomes (diurnal cohorts 3 and 8) are expressed in the morning and are preferentially up-regulated by negative DNA supercoiling, while night-expressed genes are supercoiling-repressed (cohort 2) or AT-rich (cohort 7). We find no general correlation of our AT2 periodicity clustering of CDS to this diurnally co-transcribed gene cohorts (Figure 4D), with the exception of diurnal cohort 5 and cohort 11 (genes that were not on the microarray).

Both cohorts 5 and 11 comprise several distinct transposons and unknown proteins (Supplementary Figures S11B and S13A) and have an unusually high AT content (Supplementary Figure S12B), typical of genes of foreign origin (60,61). Cohort 5 is characterized by low amplitude diurnal transcript profiles in-phase with the supercoiling-activated and GC-rich growth genes (cohorts 3 and 8, Figure 4C). The underlying genes are the same multiple copies of the ISY100 transposase (separately annotated 5′ and 3′ halves) that also underlie the enrichment of CDS periodicity clusters at *T*_max_ 11.1 and 12.5 bp with transposase annotations (Supplementary Tables S1 and S2). However, both AT2 periodicity and diurnal expression are diverse for the set of 120 annotated transposons in *Synechocystis* sp. PCC 6803 (Supplementary Figure S13). Transcription of a transposase gene depends on the genomic context into which it has been transposed, and it can not be judged whether the observed AT2 periodicity of ISY100 is causally related to its diurnal transcript profiles.

### Transposon curvature

**Figure 5.**
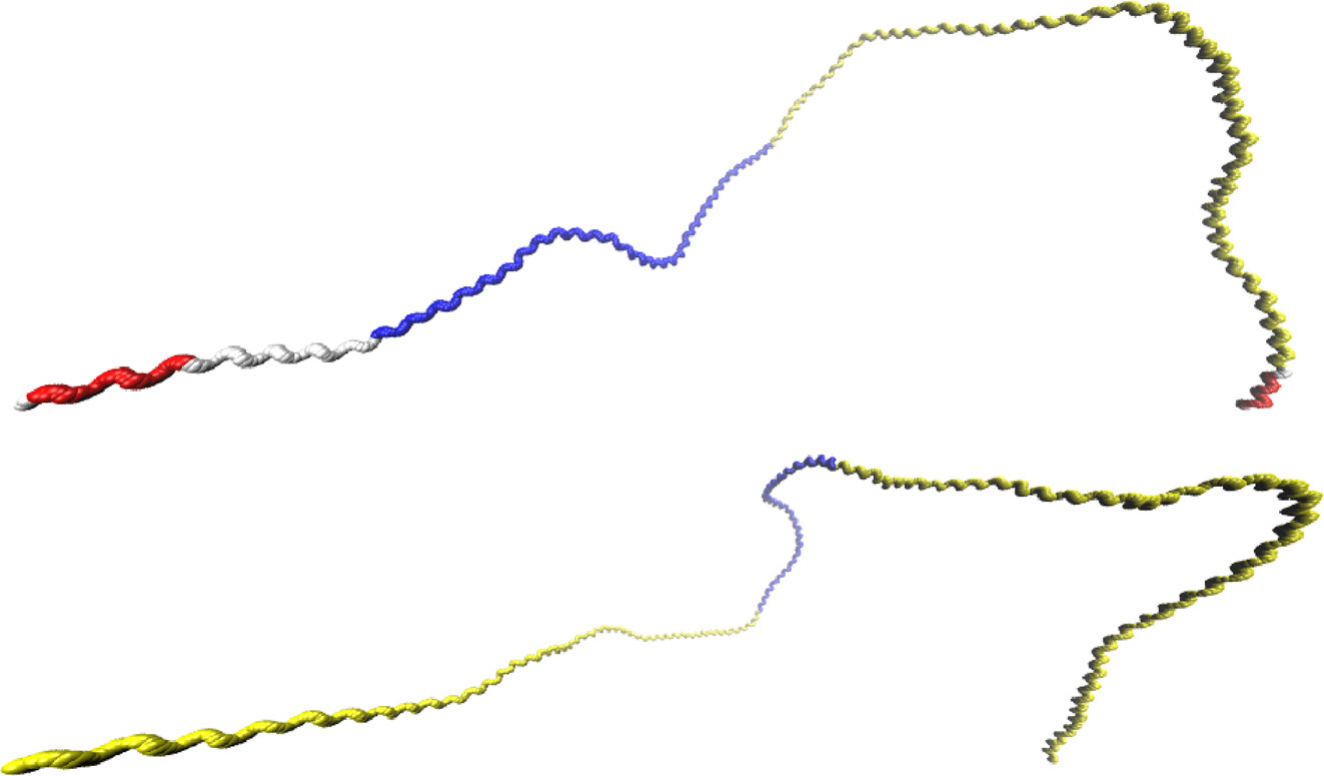
Transposons curvature. The DNA curvature paths of the ISY100f (*slr0230*) transposon of *Synechocystis* sp. PCC 6803 (top, 951 bp) and the PCC8801_2977 tranposase ORF of *Cyanothece* sp. PCC 8801 (bottom, 1227 bp) were predicted with the webserver model.it (66) using the parameter set from (52) and visualized in VMD (67). Single nucleotides of the coding strand are shown as ‘beads’, the 5′ end is on the left. For ISY100f the inverted terminal repeats are included (68) and shown in red, the annotated Pfam domains PF01710 (ISY100f, amino acids 1–111) and PF01610 (PCC8801_2977, amino acids 157–255) are shown in blue and the remaining ORF in yellow.

Intrinsic DNA curvature has been suggested to play a role in plectonemic DNA looping in both transpososome formation (63,64) and transposon silencing (65). Multiple copies of distinct transposon families in *Synechocystis* sp. PCC 6803 and *Cyanothece* sp. PCC 8801 carry specific AT2 periodicity profiles in our clustering (Supplementary Table S1). Visual inspection of the nucleotide sequences of representative genes of these CDS clusters (ISY100f/*slr0230* and PCC8801_2977) reveals an abundance of A- and T-tracts often extending over 3–4 codons (Supplementary Figures S14 and S15). To analyze DNA curvature of these transposons directly and independently from our periodicity measures we calculated a predicted DNA curvature path with the webserver tool model.it (66) using dinucleotide twist, roll and tilt angles estimated from electrophoretic mobility anomalies of synthetic DNA fragments (52), and visualized the curvature in VMD (Visual Molecular Dynamics, 67) showing reported (68) and annotated structural features (Figure 5). While such dinucleotide models are problematic for predicting sequence-dependent behavior of the DNA helix (53), they can reflect a coherent curvature of the helix by helically phased biases in dinucleotide composition. Both sequences show extensive and coherent long-range curvatures over the complete 3′ halves (*T*_max_ = 11.1 bp for the ISY100f 3′ half and the PCC 8801 proteins) and a shorter range bend within mostly linear 5′ halves (T_max_ = 12.5 bp in ISY100) of the transposase open reading frames (ORF). The 3′ halves reflect the reported curvature of *Drosophila mauritiana*'s Mos1 transposon (64) which belongs to the same superfamily of Tc1/mariner/IS630 transposons as ISY100.

## DISCUSSION

### AT2 periodicity as a ‘Structural Code’?

Among all dinucleotides, the combinations of W (A or T) give the strongest genome-wide signal at periods 10–12 bp in 74% of the 58 tested genomes, and specifically the AT2 motif in 60%. Only a few picocyanobacteria and the symbiotic cyanobacterium UCYN-A (*Candidatus Atelocyanobacterium thalassa*) have completely lost or strongly reduced AT2 periodicity at ∼11 bp (Figure 2, Supplementary Figures S1 and S2–S6). Coding regions are the main source of the AT2 signal in species with very pronounced genome-wide signals (Figure 3 and Supplementary Figures S2–S6). But even in species where the signal is comparatively weak, it is harbored most prominently at the synonymous codon position III (11,33), which contributes in combinations with position II of the same or position I of the next codon. Position II permutations have overall the weakest but still substantial effects on the signal. Both, the amino acid order of proteins and their codon usage, are partially adapted to encode this putative DNA structural information.

The AT2 motif is a minimal unit of AT-tracts. Intrinsic DNA curvature induced by phased AT-tracts leads to their localization in the apices of plectonemes of negatively supercoiled DNA (13–16). Our observations are fully consistent with the previous interpretation of phased AT-tracts in *E. coli* as a ‘structural code’ which may direct nucleoid condensation ‘in a pre-arranged manner’ (32) (hypothesis H1B in Figure 1A).

#### Relation to RNA transcription

The curvature of intergenic regions, and specifically its role in initiation or termination of RNA transcription, has been extensively analyzed (18,20,21,23,24,59). While we observe significant enrichments of 200 bp windows of pronounced AT2 periodicity in intergenic regions of weakly periodic genomes, a high genome-wide signal stems from a much stronger association of ∼11 bp periodicity with protein coding regions. Curvature downstream of a promoter may still influence gene expression, e.g. by sequestration of promoters into plectonemic DNA loops (19,60,61), but we find no comprehensive relation of the AT2 signal in coding regions to supercoiling-sensitive and diurnal transcription in *Synechocystis* sp. PCC 6803 (Figure 4, Supplementary Figures S11 and S12). Hypothesis H1A (Figure 1A) can, however, not be fully excluded by the presented analyses: the relation may depend on long-range interactions, chromosomal domain architecture (69,70) or operon structure (71). RNAseq-based transcriptome data may refine this result in future studies.

#### Multiple DNA-structural ‘Codes’

Our main measure, AT2 periodicity at ∼11 bp, likely reflects only one of many DNA-structural features encoded in bacterial genomes. Distinct periods may underlie distinct structures, such as plectonemic (>10.5 bp) versus solenoidal (<10.5 bp) DNA conformations (12). Our high-resolution scan of genomes revealed additional genomic segments with AT2 periods ∼10 bp in highly periodic species, and enrichment in intergenic regions of both ∼10 and ∼11 bp period ranges in weakly periodic genomes (Figure 3A and Supplementary Figure S8K), consistent with previous observations (9,23,24,32). However, DNA curvature can also be achieved by other nucleotide combinations (52). Alternating pyrimidine/purine stretches support transition to Z-DNA. Abundances of curved DNA and Z-DNA forming patterns are associated with general lifestyle features, such as pathogenicity, growth temperature and oxygen requirement (72,73). Studies on the relative abundances of distinct sequence patterns are becoming available (73). Analyses of their relative localizations within genomes will be required for an integrated understanding of the multiple DNA structural codes.

### AT2 periodicity in cyanobacteria

Why is the AT2 signal at ∼11 bp more pronounced in some cyanobacterial genomes? We observed (Figure 2 and Supplementary Figure S7) an exceptionally high signal in the SPM clade of unicellular cyanobacteria (morphological section I), intermediate in the heterocyst-forming clade Hcyst (section IV) and weak in picocyanobacteria (clade Syn/Pro, section I). The enterobateriaceaeal reference species (*E. coli* and *D. dadantii*) carry comparably weak but well detectable AT2 periodicity. These trends and especially their exceptions provide clues toward a more specific functional interpretation. We found several independent events, where loss (or gain) of high genome-wide AT2 periodicity is accompanied by lifestyle transitions, specifically by changes of cellular proliferation modi.

#### High AT2 periodicity

Several species of the SPM/Hcyst sister clades and *S. elongatus*, at the base of the Syn/Pro clade, are well known for oligoploid or polyploid lifestyles, where DNA replication is coupled to cell growth but not to cell division (74–79). In *Synechocystis* sp. PCC 6803 multiple chromosome copies are segregated randomly to the two daughter cells, very late during the cell cycle, and most likely by a passive process through the closing of the division septum (74). In *S. elongatus* PCC 7942 genome replication is also asynchronous and uncoupled from cell division but segregation is less random (77,78). Individual genome copies transiently align along the long axis of the cell (78), interspersed with carboxysomes (79). In stark contrast to this, DNA replication is strictly coupled to chromosome segregation and cell division in monoploid model bacteria like *E. coli* or *Bacillus subtilis* (80,81). While homologs of the HU protein and the SMC chromosome condensation complex can be found in cyanobacteria, many other proteins involved in nucleoid organization and segregation are absent (74,82).

#### Weak AT2 periodicity

Several picocyanobacteria from both *Prochlorococcus* and *Synechococcus* genera showed a strict coupling of S-phase to cell division and stable ploidy (57,58,83). Picocyanobacteria may thus have evolved novel (or re-activated old) mechanisms to mediate a stringent coupling of DNA replication and cell division. Within the highly periodic SPM clade, three genomes show strongly reduced AT2 periodicity compared to their closest relatives. Two of these are species from the morphological section II: baeocyte-forming cells proliferate by a process called multiple fission, i.e. rapid division of a vegetative cell into at least four to over 100 spherical and small baeocytes that are subsequently released (54). This implies evolution of a distinct (non-random) mode of chromosome segration. The unclassified cyanobacterium UCYN-A (*Candidatus Atelocyanobacterium thalassa*) is closely related to highly periodic *Cyanothece* strains but, as a symbiont of a unicellular alga, has lost large parts of its genome and metabolic capabilities (55), and likely also differs in regulation of DNA replication and cell division.

We speculate that a pervasive ‘structural code’ can support simple modes of nucleoid packaging and random segregration in polyploid cyanobacteria, where DNA replication is coupled to growth but not to cell division. High growth rates are usually associated with increased levels of ATP-dependent and gyrase-mediated negative DNA supercoiling (62,70). Phased AT-tracts may efficiently absorb superhelical tension into plectonemic structures and thereby allow to accomodate increasing numbers of genome copies per cell with increasing growth rates.

### Transposon curvature

Two distinct transposase ORF feature AT2 periodicity at ∼11 bp and phased AT-tracts that are well recognizable in their nucleotide sequence (Supplementary Figures S14 and S15). A coherent curvature is predicted by dinucleotide-based parameter sets (52,66) (Figure 5). The ISY100 transposon belongs to the Tc1/mariner/IS630 superfamily of transposons. It is the most abundant transposon in *Synechocystis* sp. PCC 6803 (68) and its only element shown to be active. The ISY100 transposase protein can act on its DNA template both in *E. coli* and *in vitro* without any additional co-factors except for negative supercoiling of the DNA template (84). This dependence of transposition efficiency on negatively supercoiled template DNA is well documented for several transposons (85–87). Increased supercoiling can reduce the dependence on host factors, such as the DNA-bending IHF protein (88,89), which is not found in cyanobacteria. Internal sequences of eukaryotic Tc1/mariner transposons were suggested to encode for intrinsic curvature that can nucleate formation of the ‘transpososome’, the complex of plectonemically interwound DNA where the two inverted terminal repeats (ITR) are both bound by the transposase (63,64). Thus, we speculate that the suggested biophysical role of phased AT-tracts in lowering the energetic barrier of and thereby localizing plectoneme formation (16) finds a very specific biological function in transpososome formation, where the limiting step is to bring the two ITR together (87).

Proteins such as H-NS in proteobacteria or Lrs in *Mycobacterium tuberculosis* can repress transcription of transposases by packing the DNA into plectonemic structures. Both proteins bind to AT-rich DNA without AT-tracts (61). Intrinsically curved DNA is thought to lie at the apices of plectonemes, flanked by H-NS-bridged AT-rich DNA (65). Homologous or analogous proteins are not known in cyanobacteria. A localized nucleation of plectonemes by phased AT-tracts in the ISY100 ORF could in general limit the transcription or specifically also underlie the diurnal transcript profiles of ISY100 copies (Figure 4C and Supplementary Figure S11). Encoded DNA curvature in Tc1/mariner/IS630 transposons could reflect a general strategy to fine-tune distinct transposon activities (expression, transposition, target site selection) with the overall physiological state of the host cell (growth, stress) *via* dependence on negative DNA supercoiling.

Strong periodicity was previously observed in transposons near centromers in *Arabidopsis thaliana* (90). The signal was interpreted to reflect a highly regular chromatin organization (strong nucleosomes) at centromeres, suggesting a role of transposons in centromer evolution (91). A function in transposition may have preceded a subsequent re-functionalization of transposon curvature in the chromatin architecture of centromers. Such a scenario could be exemplary for the increasingly acknowledged integration of mobile element activity with host physiology and evolution (92,93).

## SUPPLEMENTARY DATA

Supplementary Data are available at NAR Online.

SUPPLEMENTARY DATA
