# Supplementary material for: The structural code of cyanobacterial genomes
Source: Nucleic Acids Res. 2014 Jul 23;42(14):8873–83. doi: 10.1093/nar/gku641 (PMC4132750; doi:10.1093/nar/gku641)
Supplement: SUPPLEMENTARY DATA [file supp_42_14_8873__index.html]

The structural code of cyanobacterial genomes — SUPPLEMENTARY DATA 

# The structural code of cyanobacterial genomes

## SUPPLEMENTARY DATA

**Files in this Data Supplement:**

- SUPPLEMENTARY DATA
- Supporting Data File 2
- Supporting Data File 1
