# Supplementary material for: The structural code of cyanobacterial genomes
Source: Nucleic Acids Res. 2014 Jul 23;42(14):8873–83. doi: 10.1093/nar/gku641 (PMC4132750; doi:10.1093/nar/gku641)
Supplement: SUPPLEMENTARY DATA [file supp_gku641_si_proof.pdf]

Supporting Information for The structural code of cyanobacterial genomes.

Robert Lehmann [1,*], Rainer Machné [1,2,*,†], Hanspeter Herzel [1]
[1]Institute for Theoretical Biology, Humboldt University Berlin, Germany
[2]Institute for Theoretical Chemistry, University of Vienna, Austria

July 19, 2014

Contents

1 **Supporting Methods** 2
1.1 Autocorrelation 2
1.2 Spectral Analysis of the Autocorrelation Function: $Q_{NN}(T)$ and Normalization 2
1.3 Whole Genome Analyses: $Q^*_{SNR}(NN)$, Clustering & Phylogeny 3
1.4 Periodicity Localization Analysis 3
1.4.1 Overlaps of Periodic Segments with Annotated Features 3
1.4.2 Periodicity in Codon Positions 4
1.5 Spectral Clusters of Protein-coding Genes 4

2 **Supporting Data Files** 5
2.1 Columns in File S1: Species Clusters, AT2 Periodicity and Lifestyle Annotations 5
2.2 Columns in File S2: *Synechocystis* sp. PCC 6803 Gene (CDS) Clusters 5

3 **Supporting Figures and Tables** 6
3.1 Genome-wide Dinucleotide Periodicities 6
3.2 CDS Periodicity Cluster in PCC 6803 und PCC 8801 19
3.3 Supercoiling-sensitive and Diurnal Transcription in PCC 6803 23
3.4 Transposons in PCC 6803 und PCC 8801 25

List of Figures

S1 Principal Component Analysis 6
S2 Genome and CDS AT2 spectra, cluster A 7
S3 Genome and CDS AT2 spectra, cluster B 8
S4 Genome and CDS AT2 spectra, cluster C 9
S5 Genome and CDS AT2 spectra, cluster D 12
S6 Genome and CDS AT2 spectra, cluster D / loss 16
S7 Species genome lengths and phylogeny 17
S8 Periodic Windows & Segments 18
S9 Clustering of CDS AT2 periodicity spectra in *Cyanothece* sp. PCC 8801 19
S10 Properties of CDS peridiocity clusters 20
S11 Diurnal co-transcription cohorts in *Synechocystis* sp. PCC 6803 23
S12 Diurnal vs. supercoiling-sensitive transcription in *Synechocystis* sp. PCC 6803 24
S13 Transposons in *Synechocystis* sp. PCC6803 25

*these authors contributed equally
†Tel: +49 030 2093 9101; Email: raim@tbi.univie.ac.at

S14 Sequence of the ISY100f transposon 26
S15 Sequence of the PCC8801_2977 transposase ORF 27

List of Tables

S1 Protein domain enrichments in CDS clusters 21
S2 Function enrichments in CDS clusters 22

1 Supporting Methods

Note, that references are continued from the main article.

1.1 Autocorrelation

We use the autocorrelation function (ACF) as detailed in Schieg *et al.* 2004 [8]: the number $N_{NN-NN}(k)$ of dinucleotide motifs NN, WW or AT2, in distance k is counted and normalized by the number of possible pairs

$$p_{NN-NN}\,(k) = \frac{N_{NN-NN}(k)}{N-k-1} \quad ,$$

to obtain the pair probability in a sequence of length N. The background probability ${p_{NN}}^2$ to observe a NN-pair due to the sequence composition is obtained from the observed NN-dinucleotide probability p_{NN}. The employed correlation measure is then the difference between the observed and the background pair probability

$$C_{NN-NN}(k) = p_{NN-NN}(k) - {p_{NN}}^2 \quad .$$

As coding sequences introduce a strong 3 bp periodicity, a much better understood observation [26–28] [94], a window smoothing of width 3 bp is applied to suppress this signal.

$$\bar{C}_{NN-NN}(k) = \frac{\sum_{i=k-1}^{k+1} C_{NN-NN}(i)}{3}$$

1.2 Spectral Analysis of the Autocorrelation Function: $Q_{NN}(T)$ and Normalization

To analyze the dinucleotide autocorrelation function (ACF) $\tilde{C}_{NN-NN}$ obtained from the entire chromosomal sequence, we calculate the power spectrum after Mrázek 2010 [9] *via* the Fourier transform as

$$Q_{NN}(T) = \left| \sum_{k=k_{min}}^{k_{max}} \bar{C}_{NN-NN}(k) \exp\left(-ik\frac{2\pi}{T}\right) \right| \quad ,$$

where k_{min} bp and k_{max} bp specify the analyzed range of the ACF and are indicated for each analysis (window $k = [k_{min}, k_{max}]$ bp) and shown, e.g., in Figure 1 of the main manuscript and Figures S2–S6. The range $k \in [1, 29]$ bp was excluded to avoid signals induced by amphibatic α-helices [5,30,31,33]. The obtained spectra are then normalized by the sum of spectral components within $[T_{min}, T_{max}]$ bp to ensure comparability between different dinucleotide patterns and between genomes and windows with varying sequence composition:

$$Q^{*}_{NN}(T) = \frac{(T_{max} - T_{min} + 1) Q_{NN}(T)}{\sum_{i=T_{min}}^{T_{max}} Q_{NN}(T)} \quad .$$

where the spectral range and resolution are specified for each analysis.

1.3 Whole Genome Analyses: $Q^*_{SNR}(NN)$, Clustering & Phylogeny

To compare dinucleotide periodicity across cyanobacterial phyla, we condense the spectra $Q^*_{NN}(T)$ of the smoothed ACF (window: $k = [30, 101]$ bp, evaluated as the discrete Fourier components over the spectral range $T = (4, 36)$ bp) to a scalar reflecting the information whether the 10–12 bp periodicity is smaller than, similar to or stronger than its periodicity in the remaining spectrum. We define the two components in the interval $T_F \in (10, 12)$ (10.1, 11.8) bp as foreground spectrum $Q^*_{NN}(T_F)$, while the remaining 14 spectral components ($T_B = (4, 9) \cup (14, 36)$ bp) constitute its background spectrum $Q^*_{NN}(T_B)$. We introduce a signal-to-noise ratio, the maximum of the foreground spectral power divided by the median of the background spectral power, as indicator of the periodicity strength of each dinucleotide:

$$Q^*_{SNR}(NN) = \frac{max(Q^*_{NN}(T_F))}{\tilde{Q}^*_{NN}(T_B)} \quad .$$

Principal component analysis was performed *via* the `R` function `prcomp` by a singular value decomposition of the $Q^*_{SNR}(NN)$ matrix of all 16 NN dinucleotide combinations in all analyzed genomes (Fig. S1). Due to the prominent role of W nucleotides, the WW and AT2 motifs were included in hierarchical cluster analysis, using Euclidean distances, and 'complete linkage' for dinucleotides and Ward's minimum variance method for genomes. This clustering, cut at level $k = 4$ (Fig. 2A of the main manuscript), is provided with the strain list in Supporting File S1. Ancestral states of $Q^*_{SNR}(AT2)$ and species cluster assignments (Fig. 2B of the main manuscript) were inferred on the phylogenetic tree obtained from Shih *et al.* 2013 (based on alignments of 31 conserved proteins, [40]) using maximum-likelihood methods *via* the `R` package `ape` [95]. Only the transitions A↔B↔C↔D were allowed for discrete state modeling of ancestral cluster assignments.

1.4 Periodicity Localization Analysis

The chromosomal sequences were divided into non-overlapping windows, and $Q^*_{AT2}(T, i)$ of the smoothed ACF (window: $k = [30, 100]$ bp) was calculated for each window i as above, but for equidistant (0.2 bp) periods densely sampled over $T \in [2, 30]$ bp. While the accuracy of the spectral analysis improves with increasing window size, application of large genomic windows is problematic since periodic regions are generally only ≈100 bp in length [5,9,32,33]. Thus, we applied the small primary window size of 200 bp for the spectral ACF analysis and, where indicated (`200Avg4`), averaged over the spectra of four consecutive windows (Welch's method), resulting in adjacent 800 bp windows with 600 bp overlaps.

To exclude effects of sequence composition, we randomly permuted the sequence of each window i using `uShuffle` [50] with preservation of dinucleotide content, and recalculated its Fourier spectrum $Q^{*,perm}_{AT2}(T, i)$. A p-value $P_{T,i} = Pr(Q^{*,perm}_{AT2}(T, i) > Q^*_{AT2}(T, i))$ for each spectral component T was calculated from 5000 permutations and used to select significantly periodic windows. This yields a period T_{sig} which achieves the smallest p-value for each window. The spectral histograms of windows with $P_{T_{sig},i} < 0.01$ correspond well with the genome-wide spectra $Q^*_{AT2}(T)$ exhibiting maxima at ∼11 bp, but reveal additional peaks at ∼10 bp in some genomes with a high genome-wide periodicity (Fig. S8A–S8G). For genomes with a very weak genome-wide signal (Fig. S8H–S8J), no clear peak can be observed. The averaging procedure (`200Avg4`) yields a larger number of significant (but overlapping) windows with a higher signal-to-noise ratio, while the smaller primary windows provide a better resolution of the bimodal T_{sig} distribution in some species (∼10 *vs.* ∼11 bp peaks). We find no correlation between the GC content and the periodicity p-value of individual windows. Exemplary for *Cyanothece* sp. PCC 8801, both Pearson correlation coefficients between the smallest p-value $P_{T,i}$ for each window i and its GC content are very small with $r = -0.04$ (200 bp) and $r = -0.009$ (`200Avg4`).

1.4.1 Overlaps of Periodic Segments with Annotated Features

We concatenated adjacent significantly periodic windows ($P_{T,i} < 0.01$) within four period ranges to yield four distinct sets of non-overlapping periodic genome segments. Period ranges in bp were: $T \leq 9$, $9 < T \leq 10.5$, $10.5 < T \leq 12$ and $T > 12$. This procedure was applied to both, the non-overlapping

200 bp primary windows and the `200Avg4` windows (based on averaged spectra of 4 200 bp primary windows):

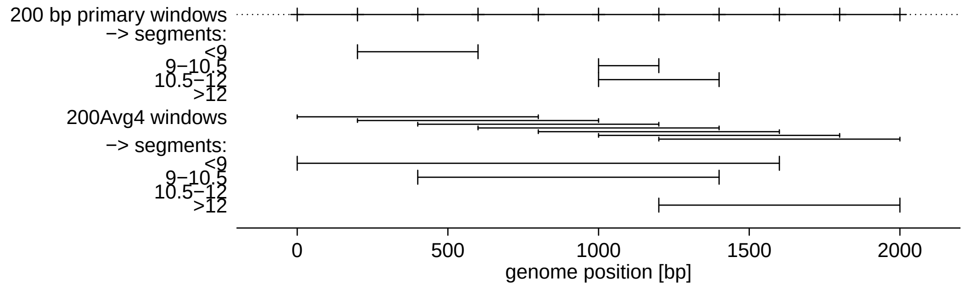

The significance of the overlaps between the obtained segments and protein-coding (CDS) and intergenic segments was tested using the Jaccard test with interval permutation as implemented in the R package `GenometriCorr` [51] (Fig. 3A of the main article & S8K).

1.4.2 Periodicity in Codon Positions

The ACF spectra $Q_{AT2}^{CDS}(T)$ were calculated from the concatenated protein-coding sequences (CDS) of each species as the discrete Fourier transform of the unsmoothed ACF (window $k = [30, 101]$ bp) and over the spectral range $T = (2, 24)$ bp. All CDS were excluded for which the annotated length is not a multiple of three. No 3 bp smoothing was performed in this analysis to compare the signal to the codon-induced 3 bp periodicity. Consequently, we also did not normalize the spectra, *i.e.* Q instead of Q^*, to avoid mutual normalization effects between the 3 bp and ∼11 bp signals. A customized version of the R package `seqinr` was used to perform codon order permutation, synonymous codon replacement (without any codon usage bias) and individual codon position permutations (with preservation of the original base composition) before concatenation of permuted CDS and calculation of the ACF spectra $Q_{AT2}^{perm}(T)$. The effects of permutations were quantified by calculating the ratios of the signal after permutation to the original signal $Q'(T) = \frac{Q_{AT2}^{perm}(T)}{Q_{AT2}^{CDS}(T)}$.

1.5 Spectral Clusters of Protein-coding Genes

The windowed periodicity measurement was applied to individual protein-coding sequences (CDS), but extending the window of the ACF to $k = [30, 130]$ bp, and evaluating the spectra at discrete Fourier components over $T = [2, 25]$ bp. CDS with less than 300 bp in length or with a dubious open reading frame annotation (*i.e.* length is not multiple of three) were excluded from this analysis. The resulting matrix $Q_{AT2}^*(T, i)$ of normalized spectral components with gene index i and period T was hierarchically clustered across genes using Ward's method, euclidean distance and the tree cut at $k = 14$ clusters. The clustering of *Synechocystis* sp. PCC 6803 CDS is provided together with the diurnally co-transcribed cohorts and supercoiling-sensitive gene groups in Supporting File S2.

2 Supporting Data Files

2.1 Columns in File S1: Species Clusters, AT2 Periodicity and Lifestyle Annotations

1. `source`: the source of the analyzed genome sequence: JGI or NCBI
2. `GenBank acc`: the GenBank accession number of the sequence
3. `taxon_oid`: the species ID at JGI/IMG
4. `name`: official NCBI name of the sequence
5. `organism`: official NCBI name of the species
6. `taxon`: the NCBI taxon ID of the species
7. `length (bp)`: genome length in basepairs
8. `GC content`: genome GC-content
9. `dinuc period cluster`: the cluster assignments (A–D) of Figure S2A
10. `AT2 SNR`: the $Q^{*}_{SNR}(AT2)$ shown in Figure 2A
11. `section`: morphological subsection obtained from the supporting material of Shih *et al.* [40]
12. `N2 fix.`: whether for which (aerobic or anaerobic) nitrogen fixation (`+`), absence of nitrogen fixation (`-`) or only of aerobic fixation (`-'`) have been shown. The information was obtained mainly from the supporting material of ref. [40] and was extended as follows: *Geitlerinema sp* PCC 7407 was positive (`+'`) in IMG "Metabolism" annotation; and *Candidatus Atelocyanobacterium thalassa* (also known as cyanobacterium UCYN-A) is described as positive for nitrogen fixation in ref. [55] (`+''`).
13. `habitat`: information from the IMG "Habitat" annotation was extended by information on "Salt tolerance" in ref. [40]: `f`: fresh water or stenohaline; `m`: marine; `a`: aquatic or euryhaline; `s`: soil; `h`: host, including *Candidatus Atelocyanobacterium thalassa* (also known as cyanobacterium UCYN-A) after ref. [55].

2.2 Columns in File S2: *Synechocystis* sp. PCC 6803 Gene (CDS) Clusters

1. `ID`: gene ID at Cyanobase
2. `name`: gene name at Cyanobase
3. `function`: gene function
4. `supercoiling_transcriptome`: supercoiling-sensitive transcript groups from ref. [49]
5. `AT2_periodicity`: cluster association in the AT2 spectral clusters of Figure 4A
6. `diurnal_transcriptome`: diurnally co-transcribed transcript cohorts as shown in Figures 4C & S11 and described in the Materials and Methods section.

3 Supporting Figures and Tables

3.1 Genome-wide Dinucleotide Periodicities

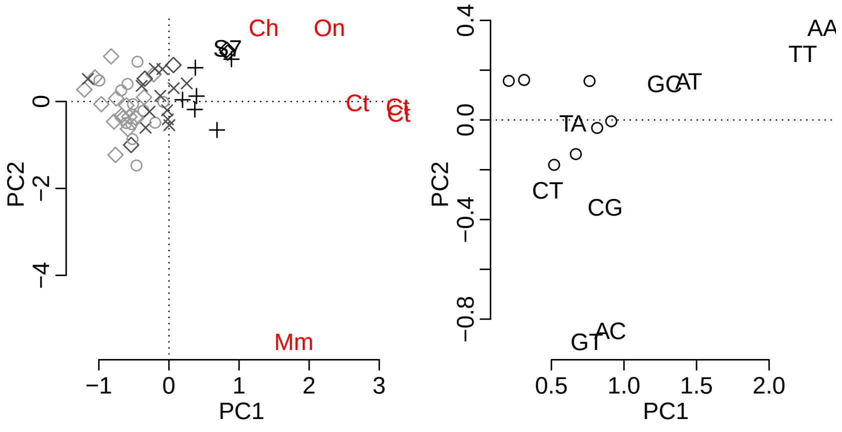

A: PCA biplot, PC1 vs. PC2

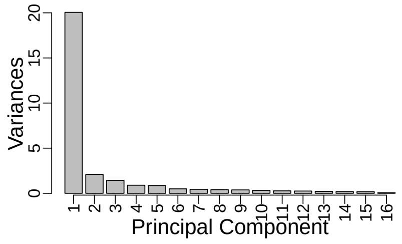

C: PCA Variances

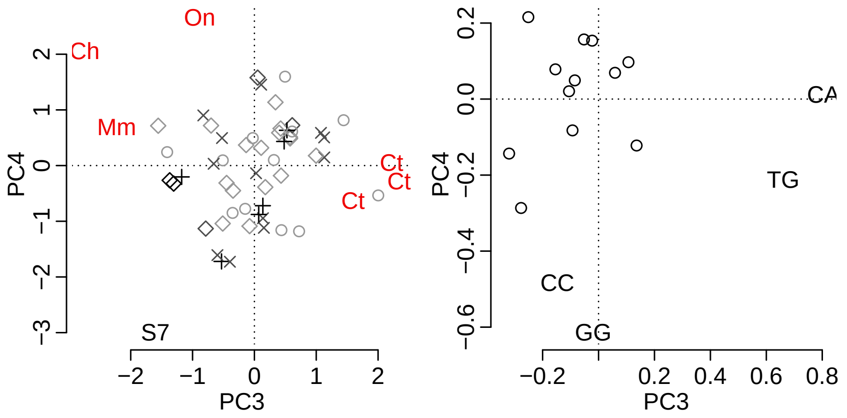

B: PCA biplot, PC3 vs. PC4

Supporting Figure S1: **Principal Component Analysis (PCA).** PCA of the $Q^*_{SNR}(NN)$ matrix shown in Figure 2A of the main manuscript, for all 16 dinucleotides NN but without the WW and AT2 motifs. Left: transformed data; colors and reflect the cluster assignment of species in Figure 2A. Plot symbols provide additional information. Diamonds: picocyanobacteria from the `Syn/Pro` clade in several clusters; light gray circles: cluster D; dark gray "x": cluster C; black "+": cluster B (S7: *Synechococcus* sp. PCC 7002); red text: cluster A, where species names are indicated by abbreviations (Mm: the Archaeum *Methanococcus marsipaludis* S2; On: *Oscillatoria nigro-viridis* PCC 7112; Ch: *Chamaesiphon minutus* PCC 6605; Ct: *Cyanothece* sp. PCC 8801, PCC 8802 and ATCC 51142). Right: rotation values for dinucleotides; only dinucleotides with strong contributions to the shown components and the TA step are plotted explicitly.

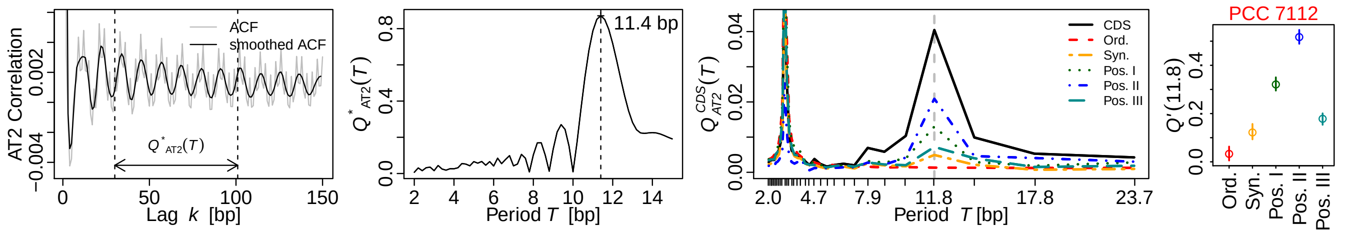

A: Oscillatoria nigro-viridis PCC 7112, cluster A, $Q^*_{SNR}(AT2)$=19.7

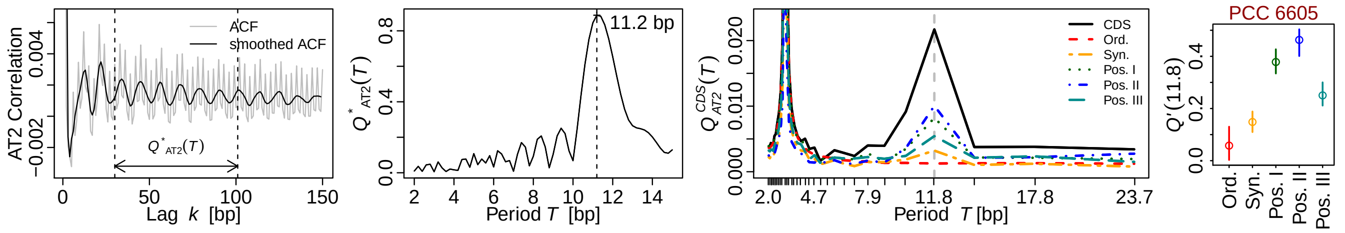

B: Chamaesiphon minutus PCC 6605, cluster A, $Q^*_{SNR}(AT2)$=15.4

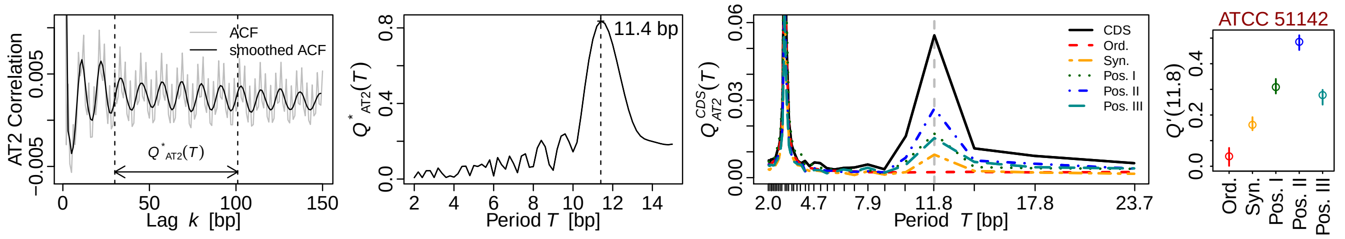

C: Cyanothece sp. ATCC 51142, cluster A, $Q^*_{SNR}(AT2)$=15.4

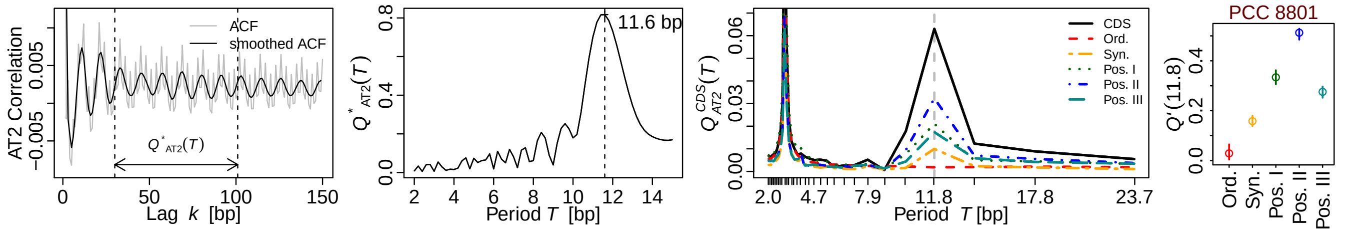

D: Cyanothece sp. PCC 8801, cluster A, $Q^*_{SNR}(AT2)$=13.8

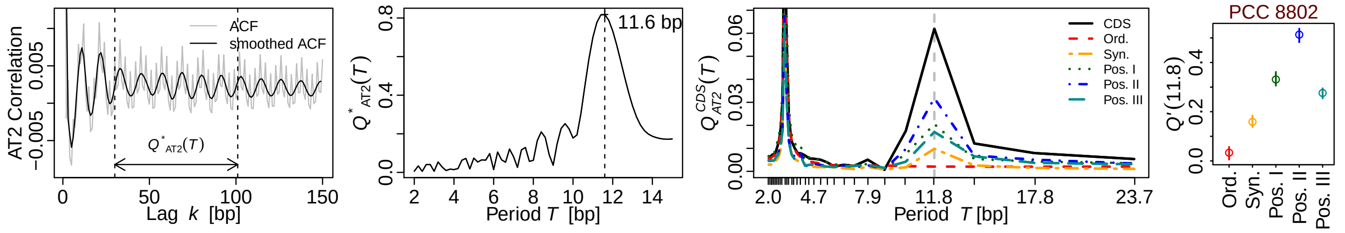

E: Cyanothece sp. PCC 8802, cluster A, $Q^*_{SNR}(AT2)$=13.6

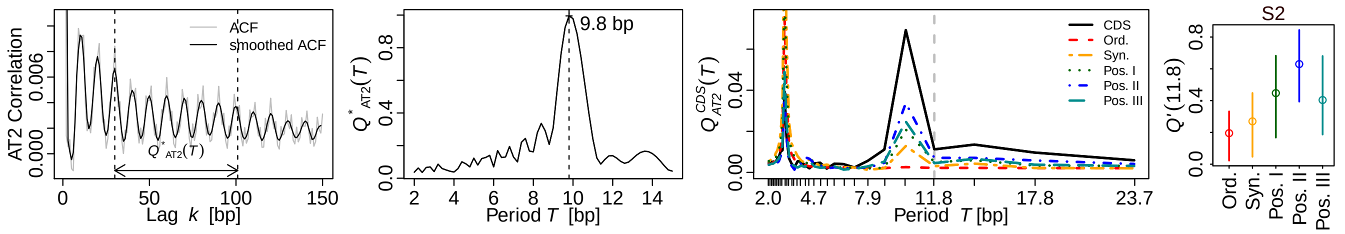

F: Methanococcus maripaludis S2, cluster A, $Q^*_{SNR}(AT2)$=11.6

Supporting Figure S2: **Genome and CDS spectra, cluster A.** Left: as Figures 1B & 1C of the main article, *i.e.* , the ACF of the AT2 motif of the complete genome and the normalized spectrum $Q^*_{AT2}(T)$ of the interval $k = [30, 101]$ bp of the smoothed ACF. The periods of the maximal spectral component in each species are indicated. Right: as Figures 3B & 3C of the main article, *i.e.* , AT2 motif spectra of all concatenated coding regions (ACF unsmoothed, unnormalized) before and after codon permutations. The spectra shown in Figures S2–S6 are ordered according to $Q^*_{SNR}(AT2)$ and the Figures are sorted by species cluster associations (*cf.* Fig. 2A of the main article). Note the peak at ~10 bp of the archaeum *Methanococcus maripaludis* S2, consistent with previous analyses [9].

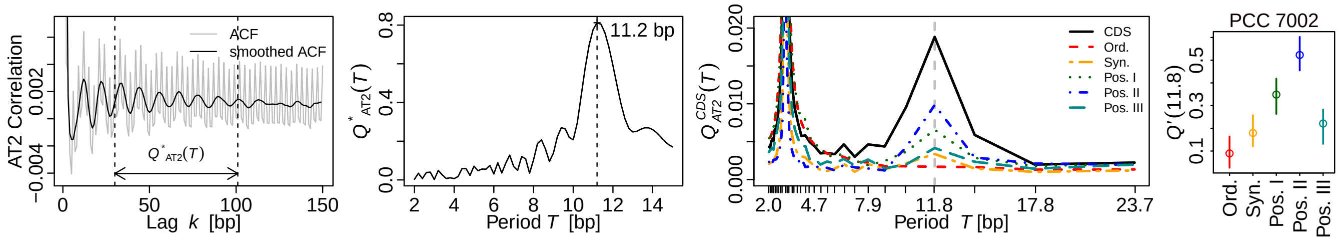

G: Synechococcus sp. PCC 7002, cluster B, $Q^*_{SNR}(AT2)$=10.5

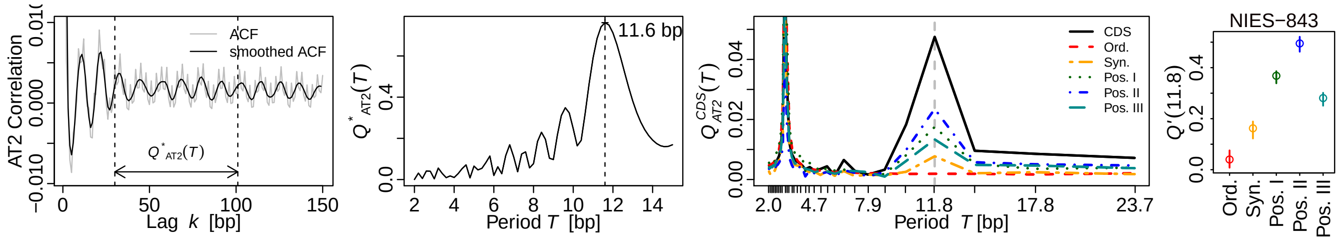

H: Microcystis aeruginosa NIES-843, cluster B, $Q^*_{SNR}(AT2)$=10.3

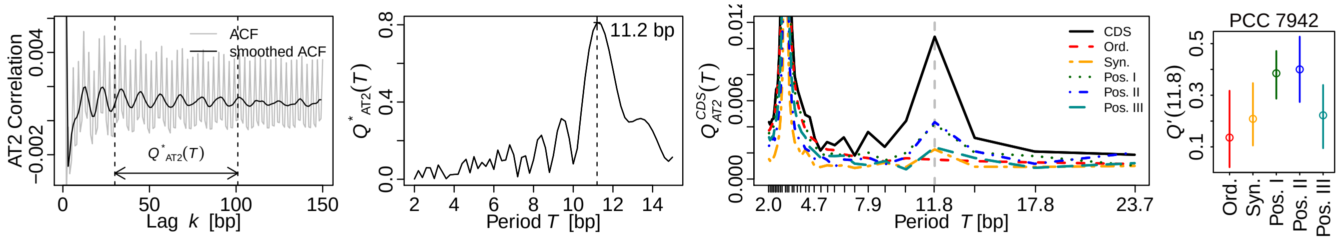

I: Synechococcus elongatus PCC 7942, cluster B, $Q^*_{SNR}(AT2)$=10.2

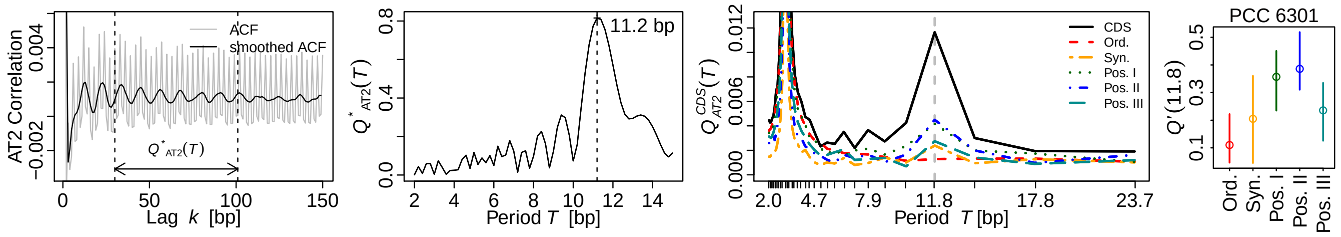

J: Synechococcus elongatus PCC 6301, cluster B, $Q^*_{SNR}(AT2)$=10.1

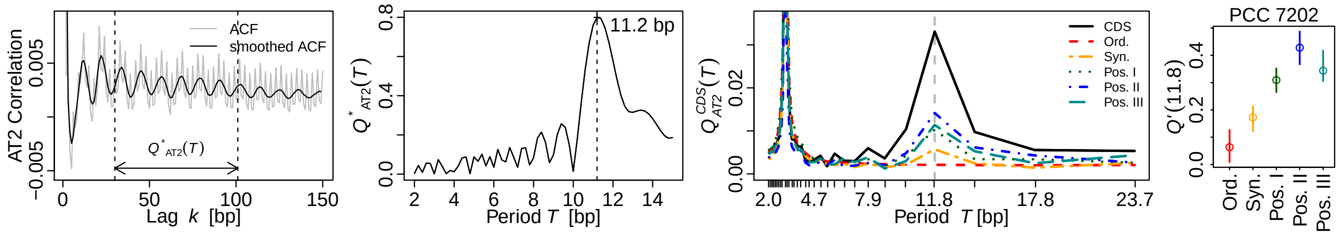

K: Cyanobacterium stanieri PCC 7202, cluster B, $Q^*_{SNR}(AT2)$=10.1

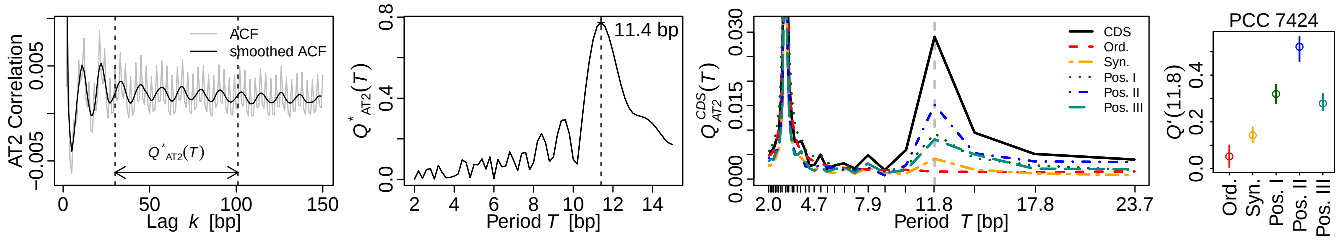

L: Cyanothece sp. PCC 7424, cluster B, $Q^*_{SNR}(AT2)$=9.6

Supporting Figure S3: **Genome and CDS AT2 spectra, cluster B.** See Figure S2 for a legend. Continued ...

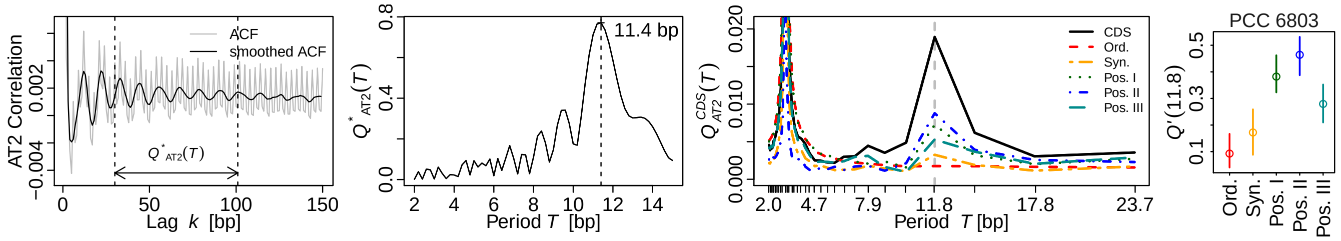

M: Synechocystis sp. PCC 6803, cluster B, $Q^*_{SNR}(AT2)$=8.9

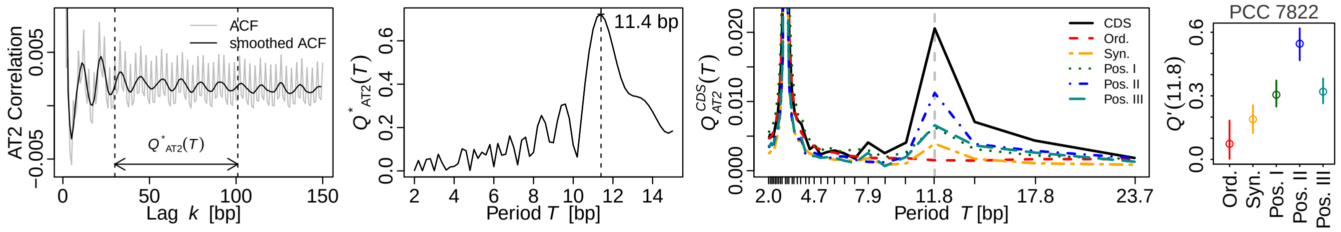

N: Cyanothece sp. PCC 7822, cluster B, $Q^*_{SNR}(AT2)$=7.8

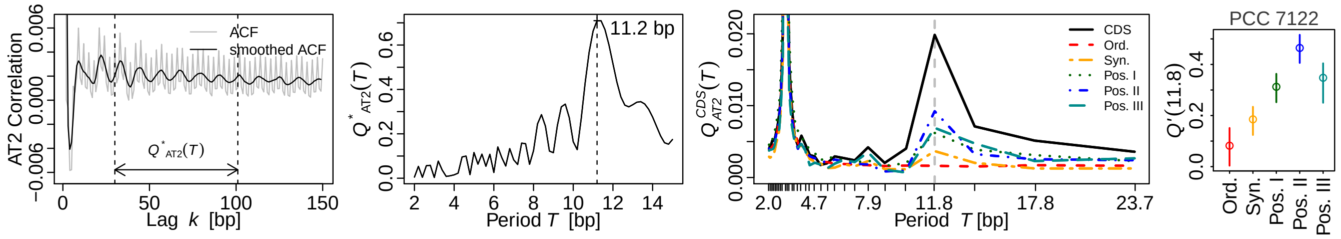

O: Anabaena cylindrica PCC 7122, cluster B, $Q^*_{SNR}(AT2)$=7.7

... continued from Supporting Figure S3, **cluster B**.

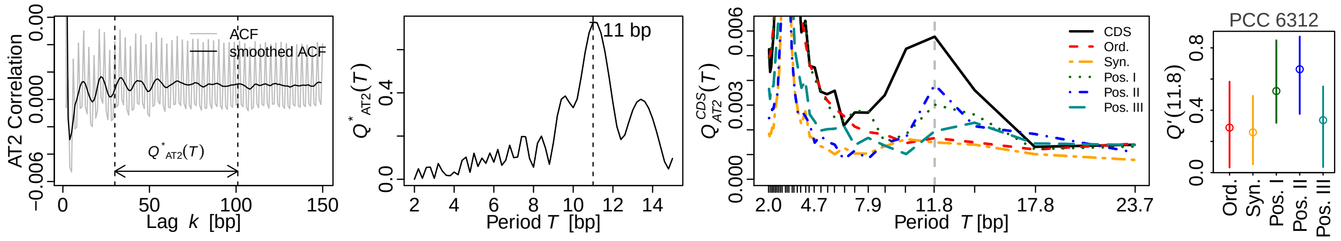

A: Synechococcus sp. PCC 6312, cluster C, $Q^*_{SNR}(AT2)$=7.5

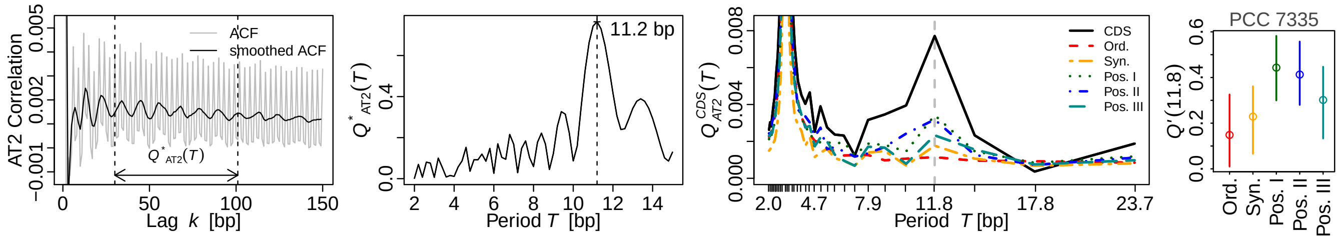

B: Synechococcus sp. PCC 7335, cluster C, $Q^*_{SNR}(AT2)$=7.1

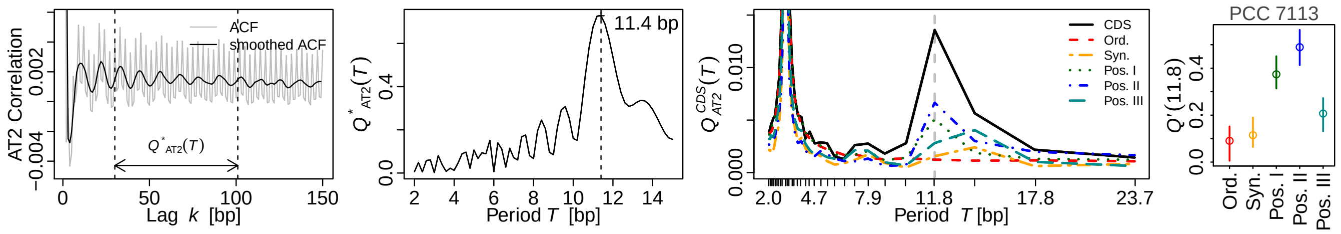

C: Microcoleus sp. PCC 7113, cluster C, $Q^*_{SNR}(AT2)$=7.1

Supporting Figure S4: **Genome and CDS AT2 spectra, cluster C.** See Figure S2 for a legend. Continued ...

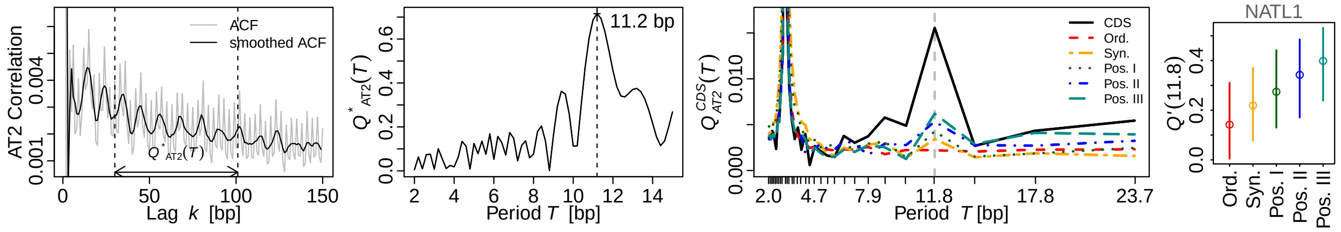

G: Prochlorococcus marinus str. NATL1A, cluster C, $Q^*_{SNR}(AT2)$=6.2

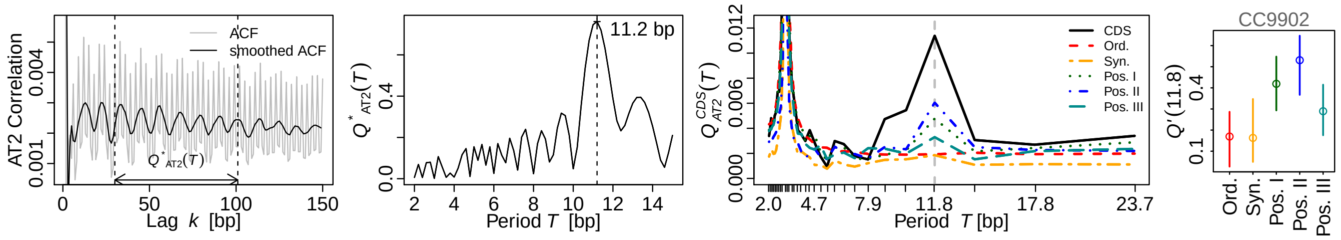

H: Synechococcus sp. CC9902, cluster C, $Q^*_{SNR}(AT2)$=6.1

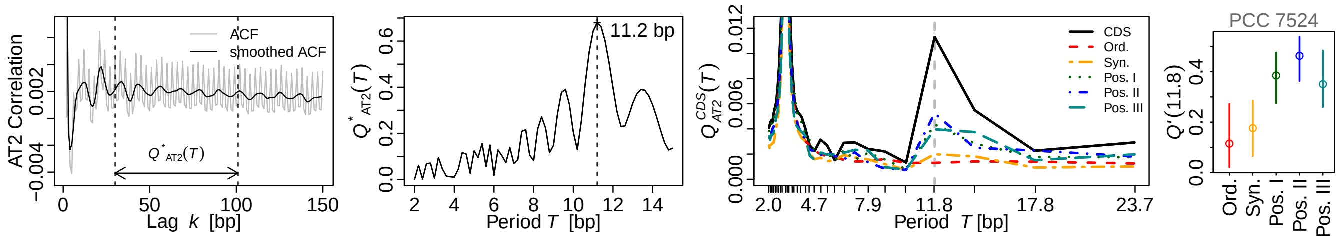

I: Nostoc sp. PCC 7524, cluster C, $Q^*_{SNR}(AT2)$=5.8

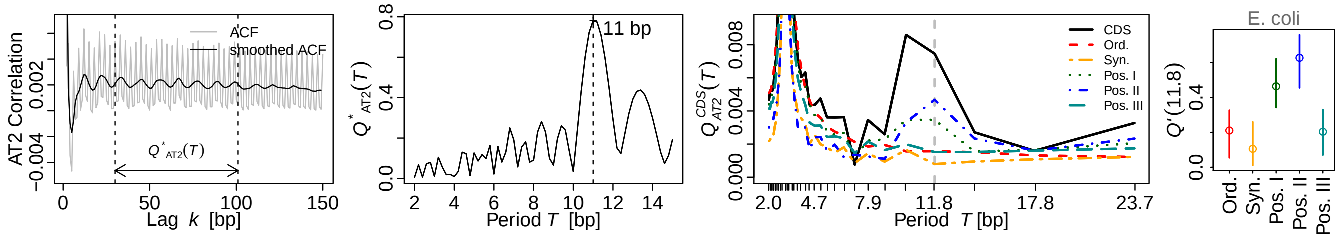

J: Escherichia coli str. K-12 substr. MG1655, cluster C, $Q^*_{SNR}(AT2)$=5.8

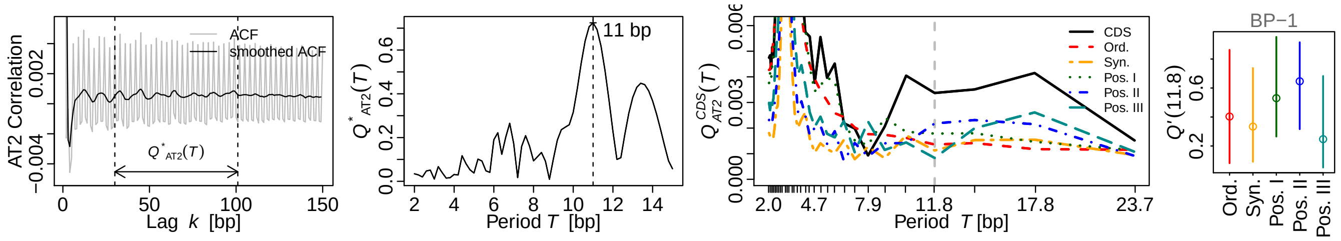

K: Thermosynechococcus elongatus BP-1, cluster C, $Q^*_{SNR}(AT2)$=5.7

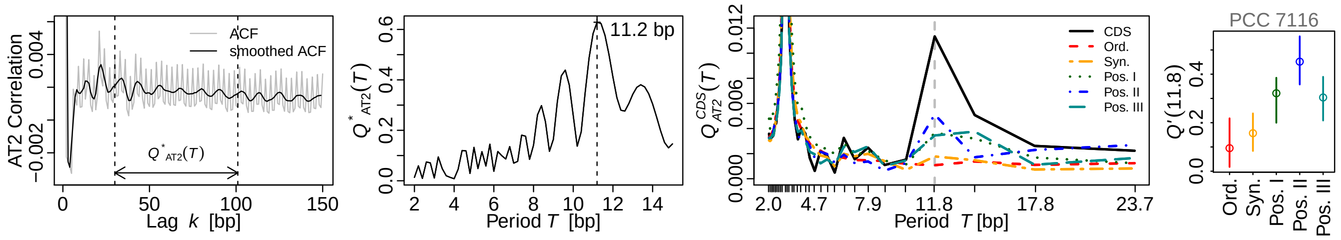

L: Rivularia sp. PCC 7116, cluster C, $Q^*_{SNR}(AT2)$=5.7

... continued from Supporting Figure S4, **cluster C**. Note the broader peak in the CDS spectrum of *Escherichia coli* despite the clear whole-genome peak at 11 bp; and the very broad peak of *Thermosynechococcus*. Continued ...

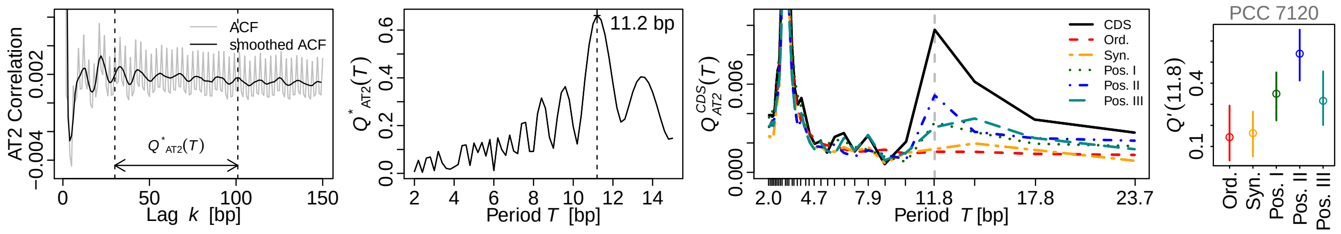

M: Nostoc sp. PCC 7120, cluster C, $Q^{*}_{SNR}(AT2)$=5.6

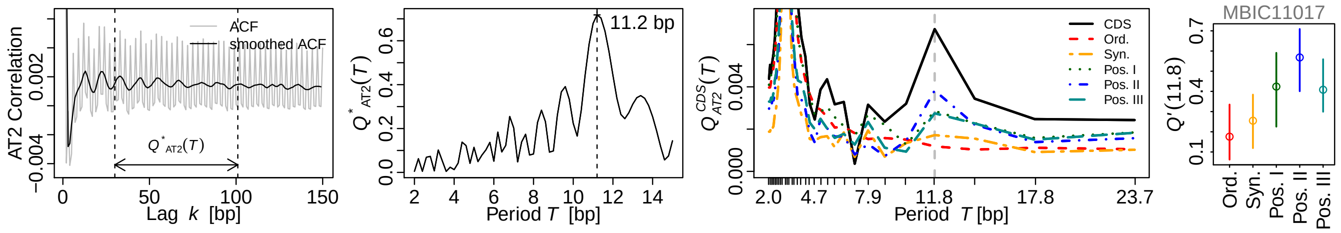

N: Acaryochloris marina MBIC11017, cluster C, $Q^{*}_{SNR}(AT2)$=5.4

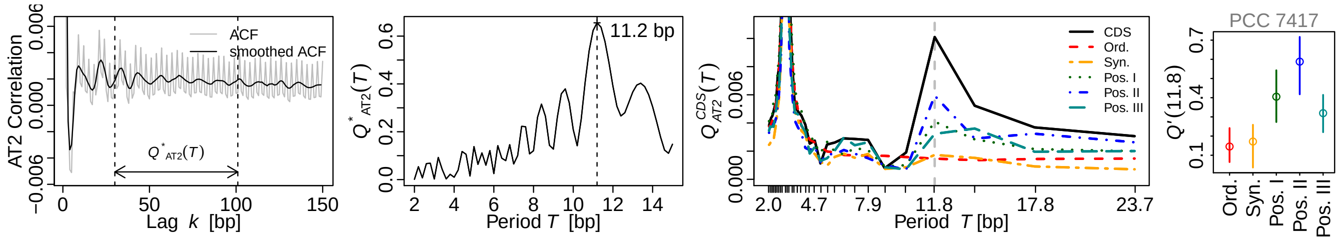

O: Cylindrospermum stagnale PCC 7417, cluster C, $Q^{*}_{SNR}(AT2)$=5.2

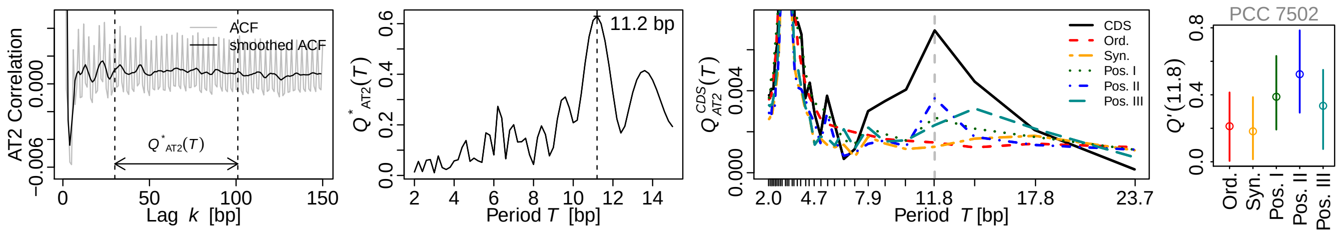

P: Synechococcus sp. PCC 7502, cluster C, $Q^{*}_{SNR}(AT2)$=4.8

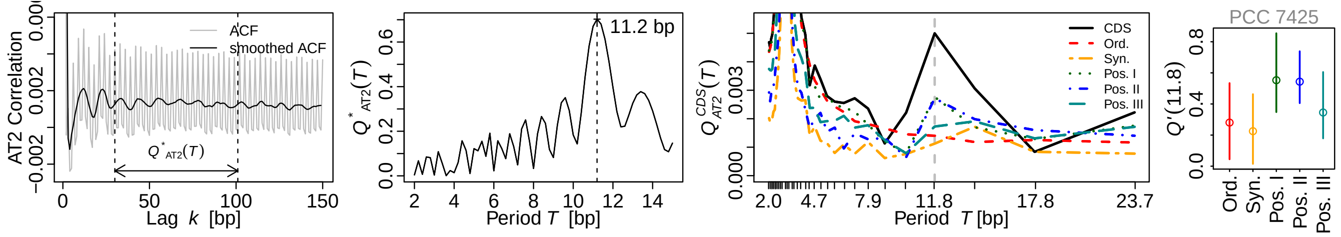

Q: Cyanothece sp. PCC 7425, cluster C, $Q^{*}_{SNR}(AT2)$=4.7

... continued from Supporting Figure S4, **cluster C**.

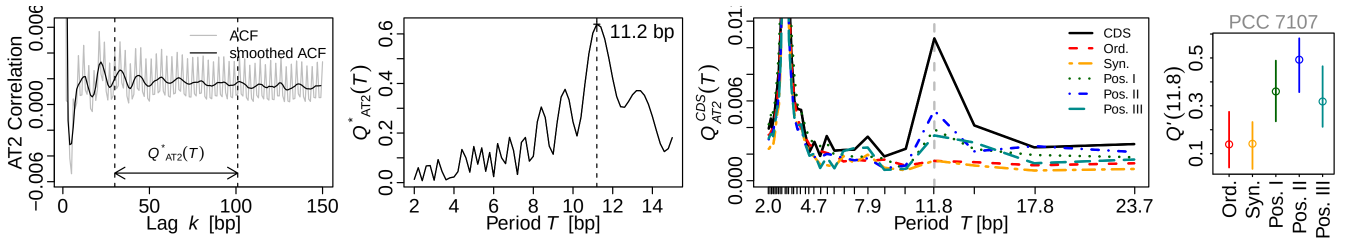

A: Nostoc sp. PCC 7107, cluster D, $Q^*_{SNR}(AT2)$=4.6

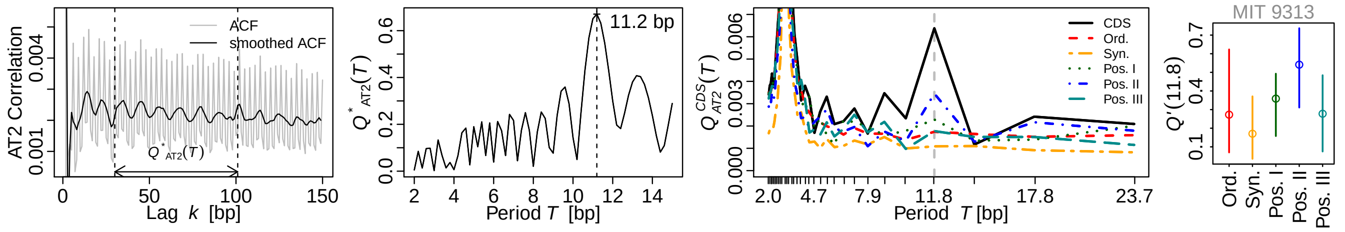

B: Prochlorococcus marinus str. MIT 9313, cluster D, $Q^*_{SNR}(AT2)$=4.4

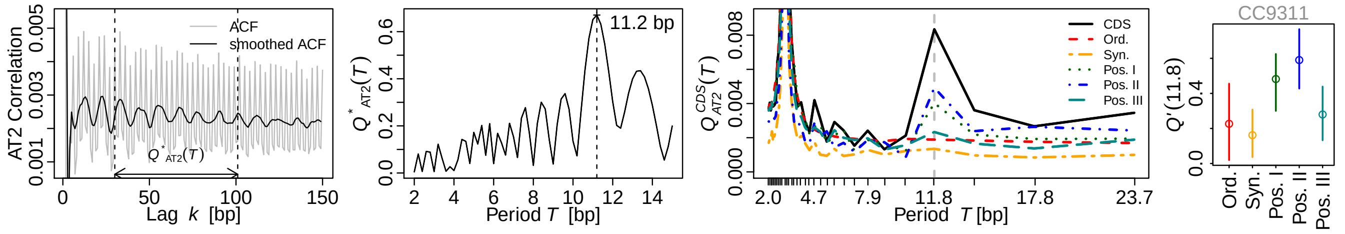

C: Synechococcus sp. CC9311, cluster D, $Q^*_{SNR}(AT2)$=4.3

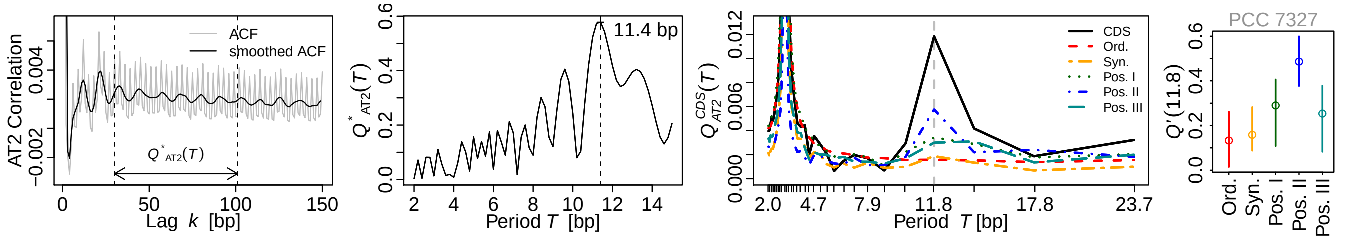

D: Pleurocapsa sp. PCC 7327, cluster D, $Q^*_{SNR}(AT2)$=4.2

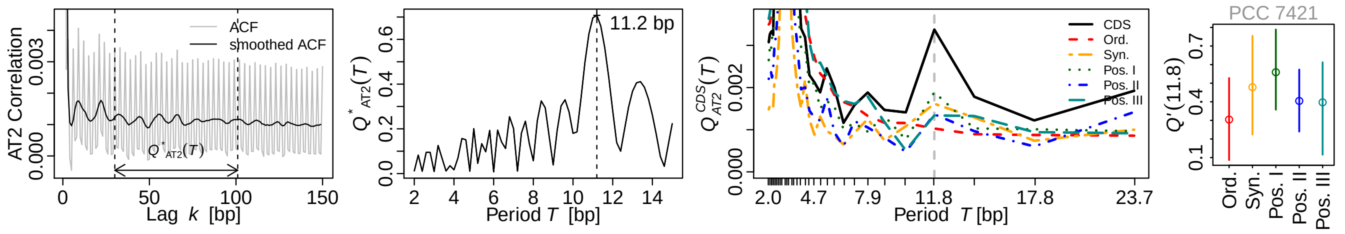

E: Gloeobacter violaceus PCC 7421, cluster D, $Q^*_{SNR}(AT2)$=4.2

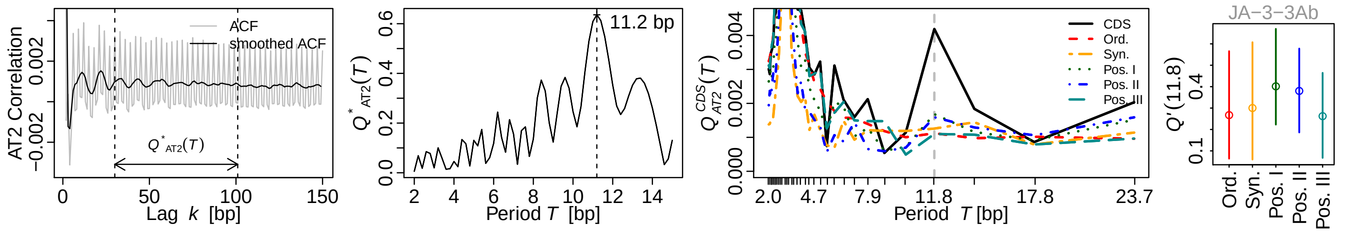

F: Synechococcus sp. JA-3-3Ab, cluster D, $Q^*_{SNR}(AT2)$=4.1

Supporting Figure S5: **Genome and CDS AT2 spectra, cluster D.** See Figure S2 for a legend. Continued ...

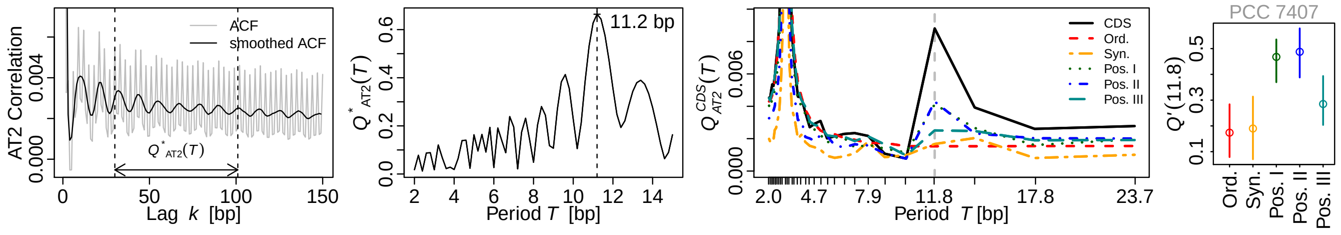

G: Geitlerinema sp. PCC 7407, cluster C, $Q^*_{SNR}(AT2)$=4.1

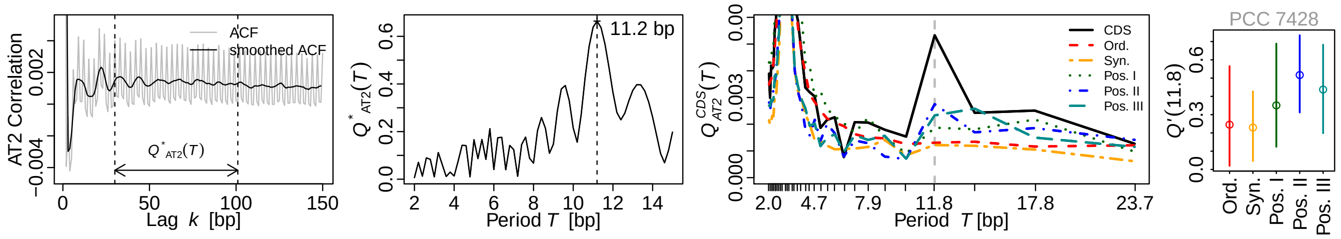

H: Gloeocapsa sp. PCC 7428, cluster D, $Q^*_{SNR}(AT2)$=4

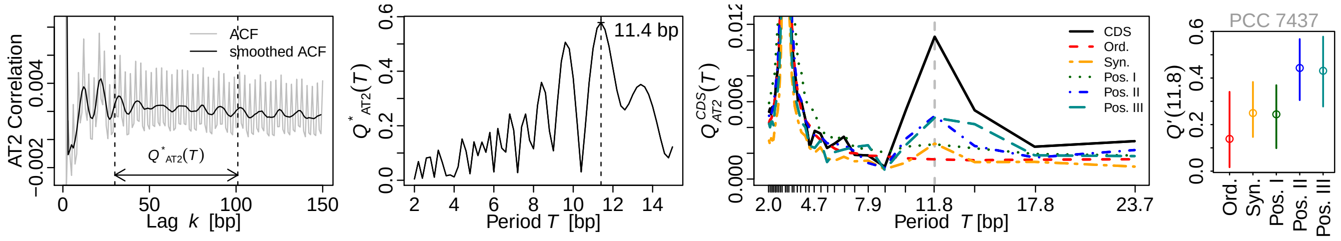

I: Stanieria cyanosphaera PCC 7437, cluster D, $Q^*_{SNR}(AT2)$=3.9

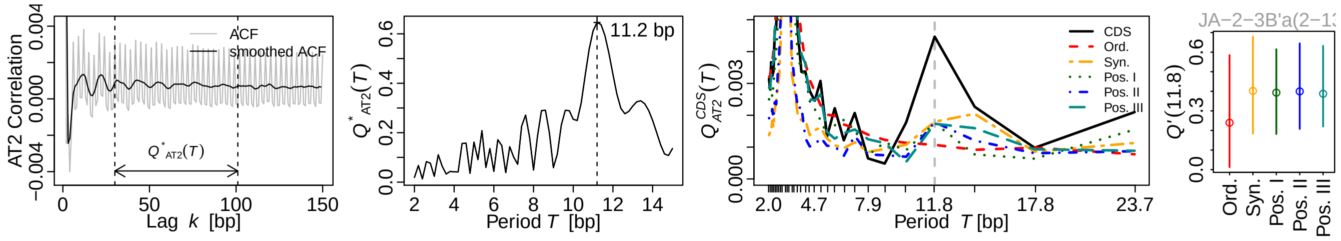

J: Synechococcus sp. JA-2-3B'a(2-13), cluster D, $Q^*_{SNR}(AT2)$=3.8

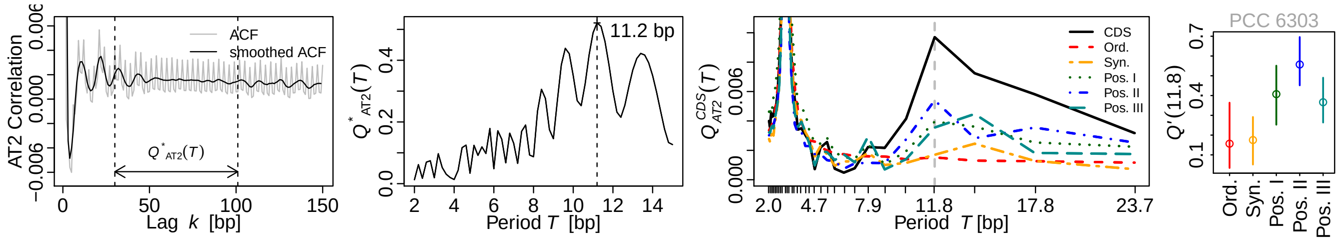

K: Calothrix sp. PCC 6303, cluster D, $Q^*_{SNR}(AT2)$=3.6

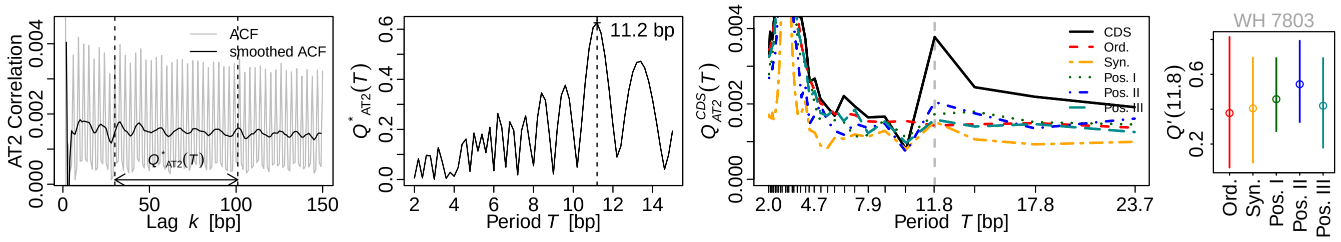

L: Synechococcus sp. WH 7803, cluster D, $Q^*_{SNR}(AT2)$=3.5

... continued from Supporting Figure S5, **cluster D**. Note *Geitlerinema* sp., cluster C, but lower $Q^*_{SNR}(AT2)$ (species are ordered by their $Q^*_{SNR}(AT2)$). Continued ...

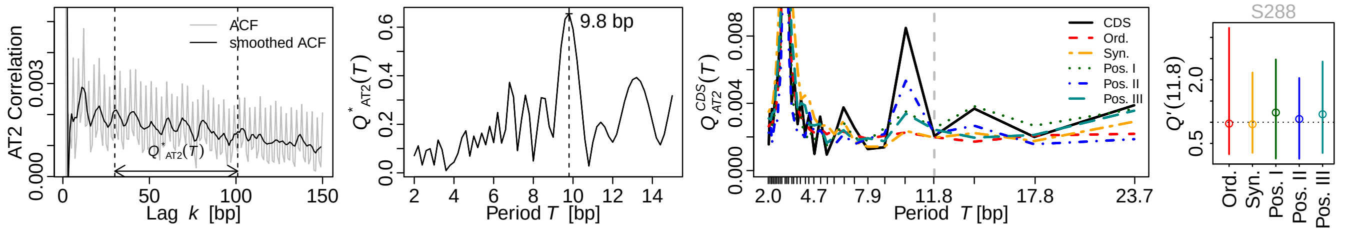

M: Saccharomyces cerevisiae S288c, cluster D, $Q^{*}_{SNR}(AT2)$=3.3

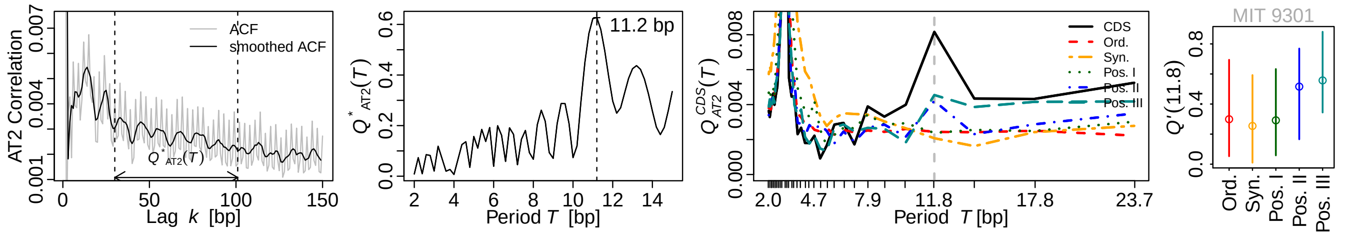

N: Prochlorococcus marinus str. MIT 9301, cluster D, $Q^{*}_{SNR}(AT2)$=3.3

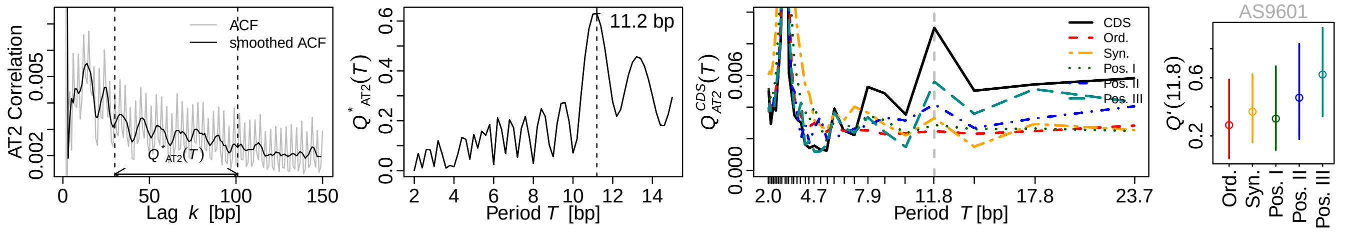

O: Prochlorococcus marinus str. AS9601, cluster D, $Q^{*}_{SNR}(AT2)$=3.3

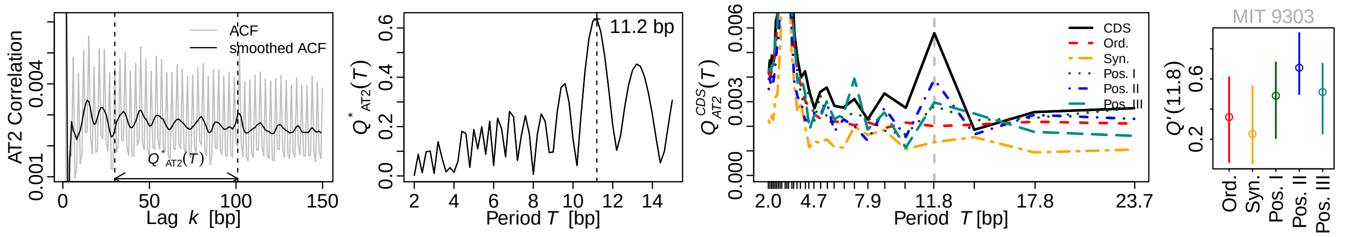

P: Prochlorococcus marinus str. MIT 9303, cluster D, $Q^{*}_{SNR}(AT2)$=3.2

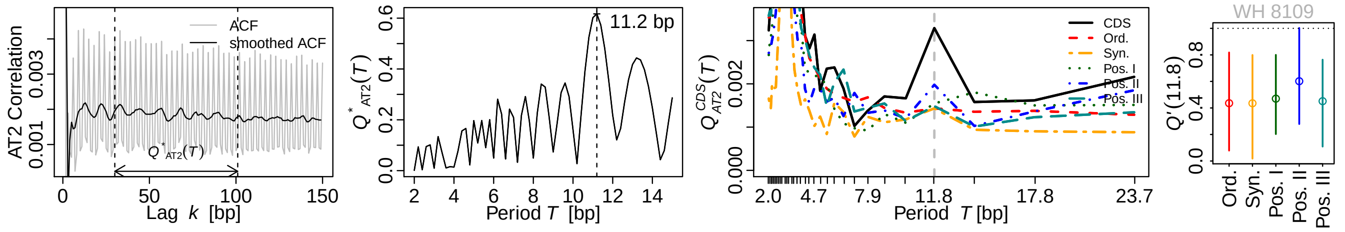

Q: Synechococcus sp. WH 8109, cluster D, $Q^{*}_{SNR}(AT2)$=3

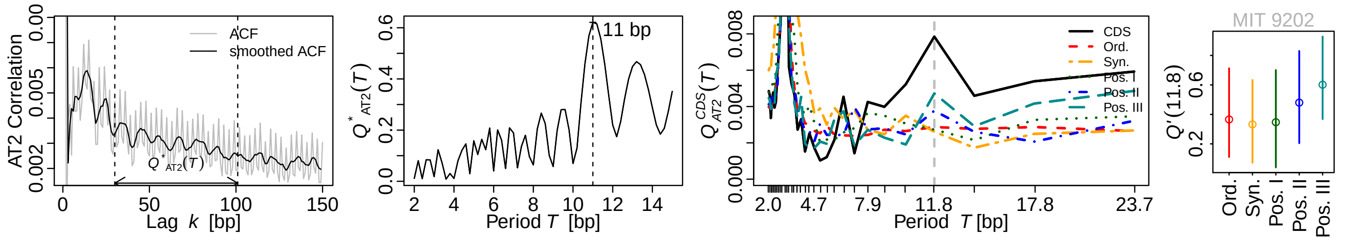

R: Prochlorococcus marinus str. MIT 9202, cluster D, $Q^{*}_{SNR}(AT2)$=3

... continued from Supporting Figure S5, **cluster D**. Note the peak at 9.8 bp in chromosome IV of *Saccharomyces cerevisiae* S288c. Continued ...

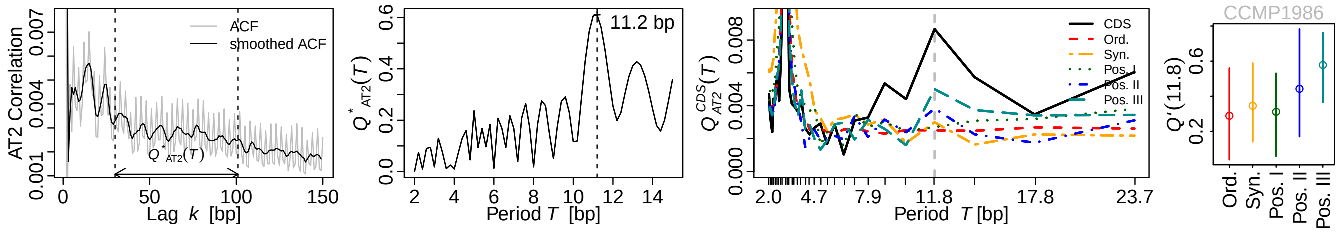

N: Prochlorococcus marinus subsp. pastoris str. CCMP1986, cluster D, $Q^*_{SNR}(AT2)$=2.8

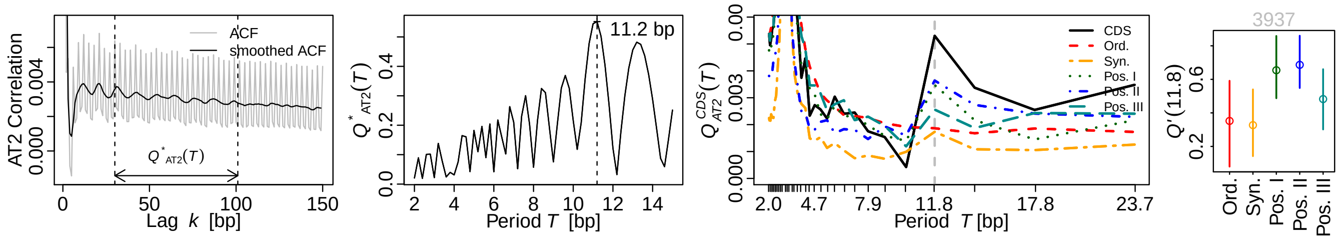

O: Dickeya dadantii 3937, cluster D, $Q^*_{SNR}(AT2)$=2.6

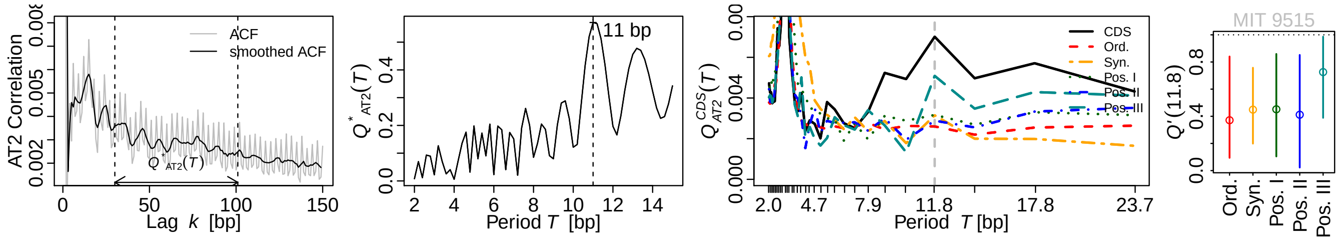

P: Prochlorococcus marinus str. MIT 9515, cluster D, $Q^*_{SNR}(AT2)$=2.5

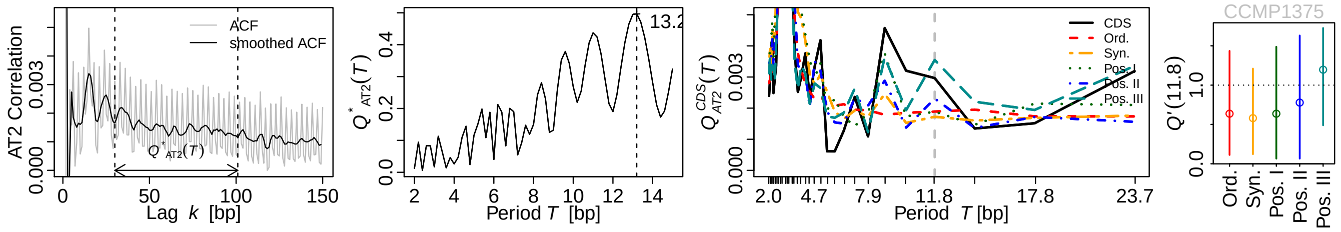

Q: Prochlorococcus marinus subsp. marinus str. CCMP1375, cluster D, $Q^*_{SNR}(AT2)$=2.5

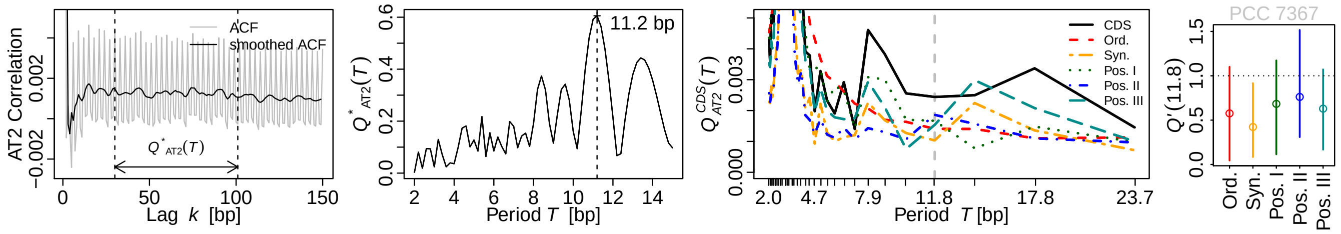

R: Pseudanabaena sp. PCC 7367, cluster D, $Q^*_{SNR}(AT2)$=2.3

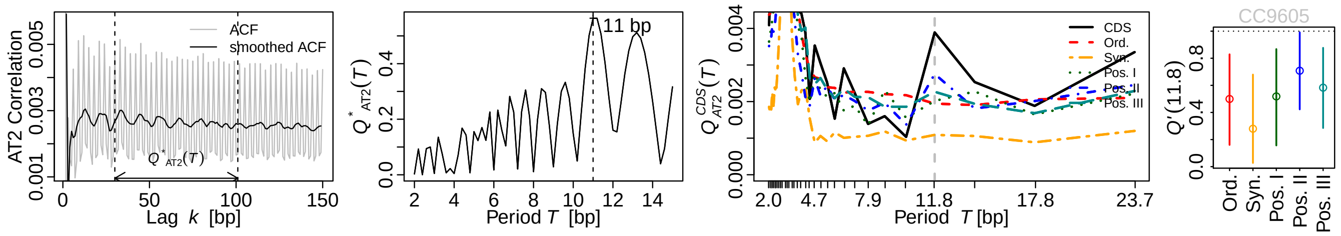

S: Synechococcus sp. CC9605, cluster D, $Q^*_{SNR}(AT2)$=2.3

... continued from Supporting Figure S5, **cluster D**. Note the degraded signals in *Prochlorococcus marinus* subsp. marinus CCMP1375, and *Pseudanabaena* sp. PCC 7367 with peaks at 8–9 bp. The latter species has still a maximum at the typical period (11.2 bp) in the genome-wide spectrum

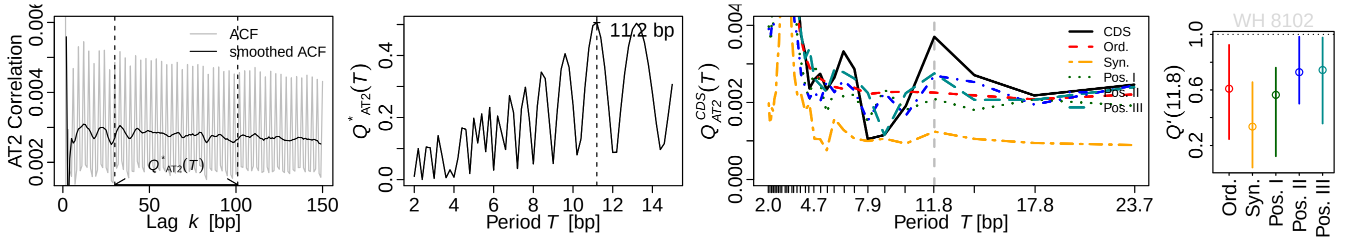

A: Synechococcus sp. WH 8102, cluster D, $Q^{*}_{SNR}(AT2)$=1.6

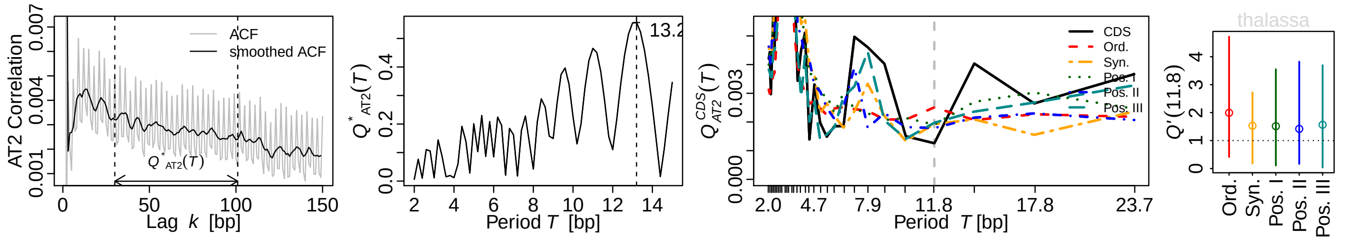

B: Candidatus Atelocyanobacterium thalassa, cluster D' / loss, $Q^{*}_{SNR}(AT2)$=1.6

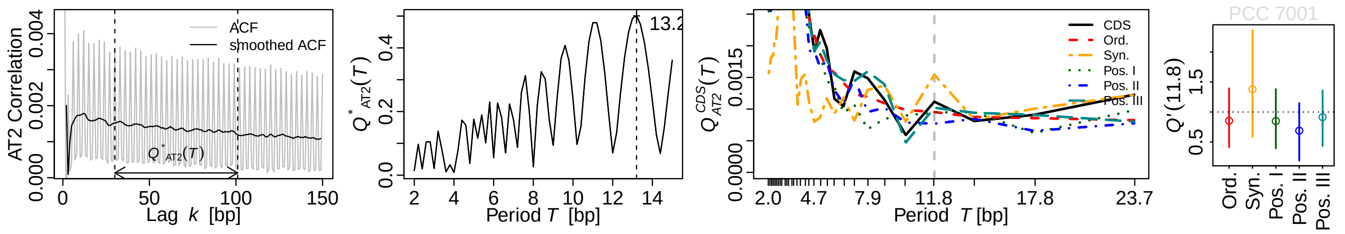

C: Cyanobium sp. PCC 7001, cluster D' / loss, $Q^{*}_{SNR}(AT2)$=1.4

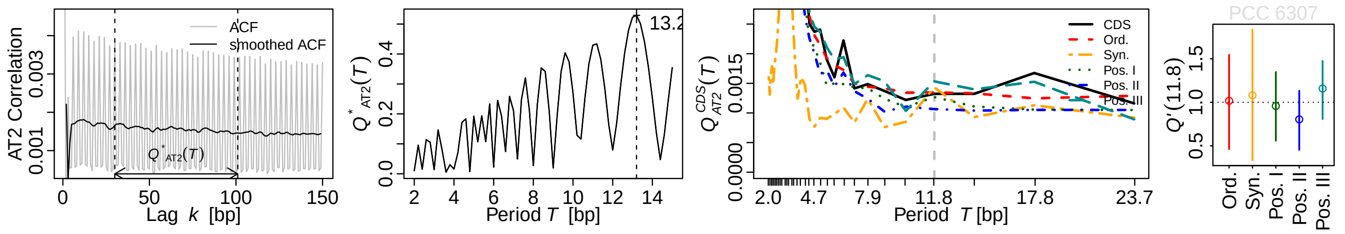

D: Cyanobium gracile PCC 6307, cluster D' / loss, $Q^{*}_{SNR}(AT2)$=1.4

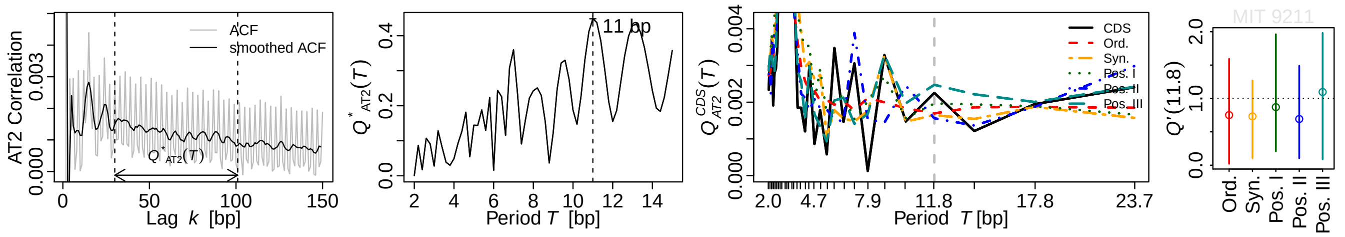

E: Prochlorococcus marinus str. MIT 9211, cluster D' / loss, $Q^{*}_{SNR}(AT2)$=1.1

Supporting Figure S6: **Genome and CDS AT2 spectra, cluster D / loss of AT2 periodicity.** See Fig. S2 for a legend. Note the shift of the genome-wide maxima to 13.2 bp and a complete loss of the ∼11 bp signal in coding regions in the four species with very weak genome-wide $Q^{*}_{SNR}(AT2)$ (species in Fig.S2–S6 were sorted by their $Q^{*}_{SNR}(AT2)$). Note that *Prochlorococcus marinus* str. MIT 9211 has a genome-wide maximum at 11 bp and still shows an increased short range auto-correlation (left panel, $k < 30$ bp) that is also seen in other *Prochlorococci* (Fig. S5) and which is excluded from our measures to control for protein-code signals.

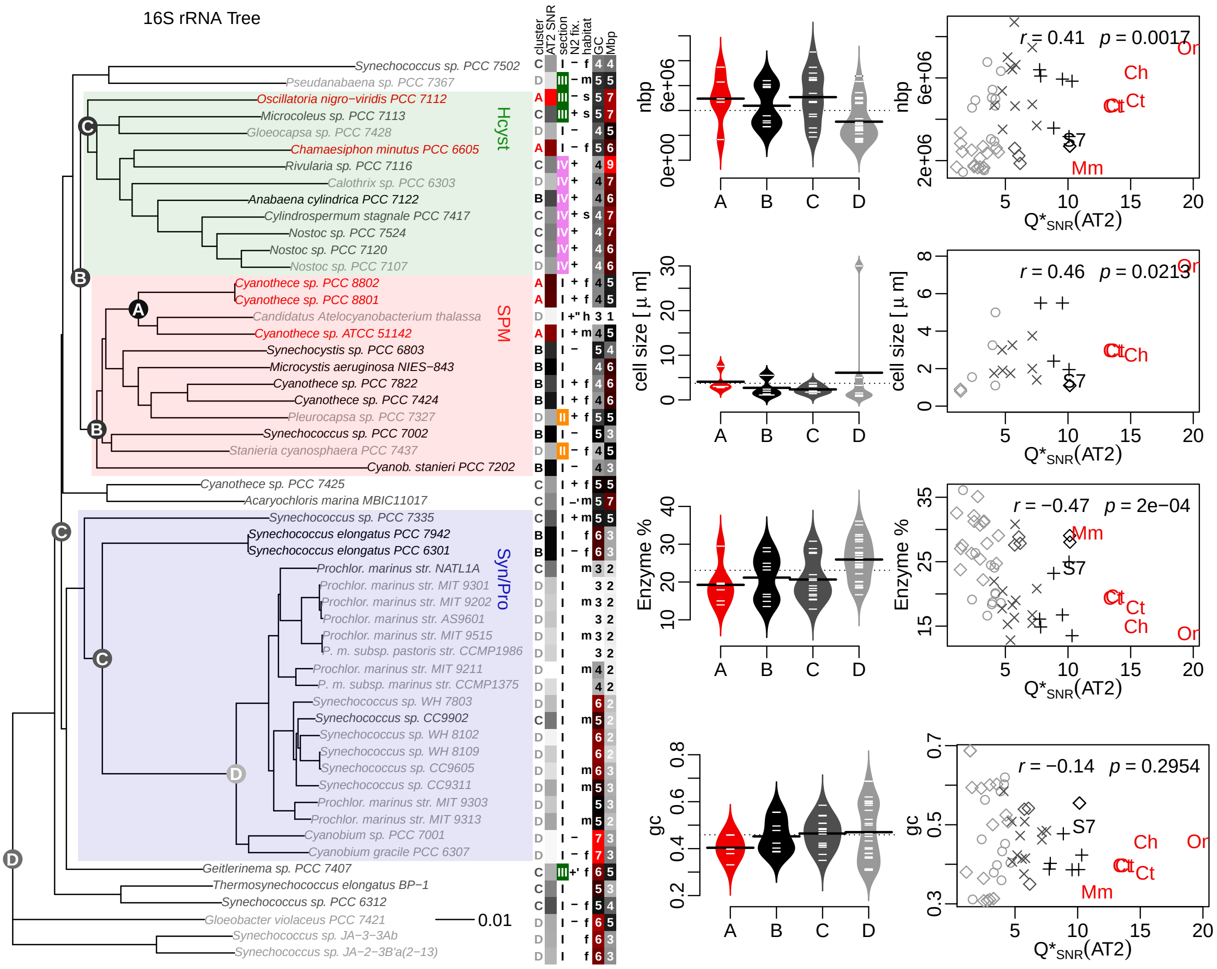

A: 16S rRNA phylogeny

B: Species/Genome Properties

Supporting Figure S7: **Species genome lengths and phylogeny.** S7A: as Figure 2B of the main article but using a phylogeny based on 16S rRNA alignments from the SILVA database [44] obtained from the IMG database [43]. Additionally the species' GC content (in %/10), and habitat annotations (from IMG and [40]; f: fresh water, m: marine, s: soil) are shown. S7B, species and genome properties: "nbp" is the length in bp and "gc" the GC content of the analyzed genomes; "cell size" is the mean of cell diameter ranges reported in the Supplemental data of ref. [40]; "Enzyme %" is the % of genes annotated als enzymes as provided at the IMG database. Left: the distributions are shown as bean-plots [96] for the species clustering in Figure 2A of the main article. Right: values are plotted against the species' $Q^*_{SNR}(AT2)$ (Fig. 2A). Pearson correlations (r, p-value) were calculated only for eubacterial species and the outlier species in cell size (*Stanieria cyanosphaera* PCC 7437 with 30 μm) was further excluded from this test. Species cluster memberships are indicated by colors and plot symbols as in Figure S1. **Summary:** the picocyanobacterial clade (`Syn/Pro`, diamonds) is characterized by small average cell size and stream-lined genomes that still encode for a "minimal oxyphototroph lifestyle" and have a higher fraction of metabolic genes (*i.e.* less regulatory proteins) [56].

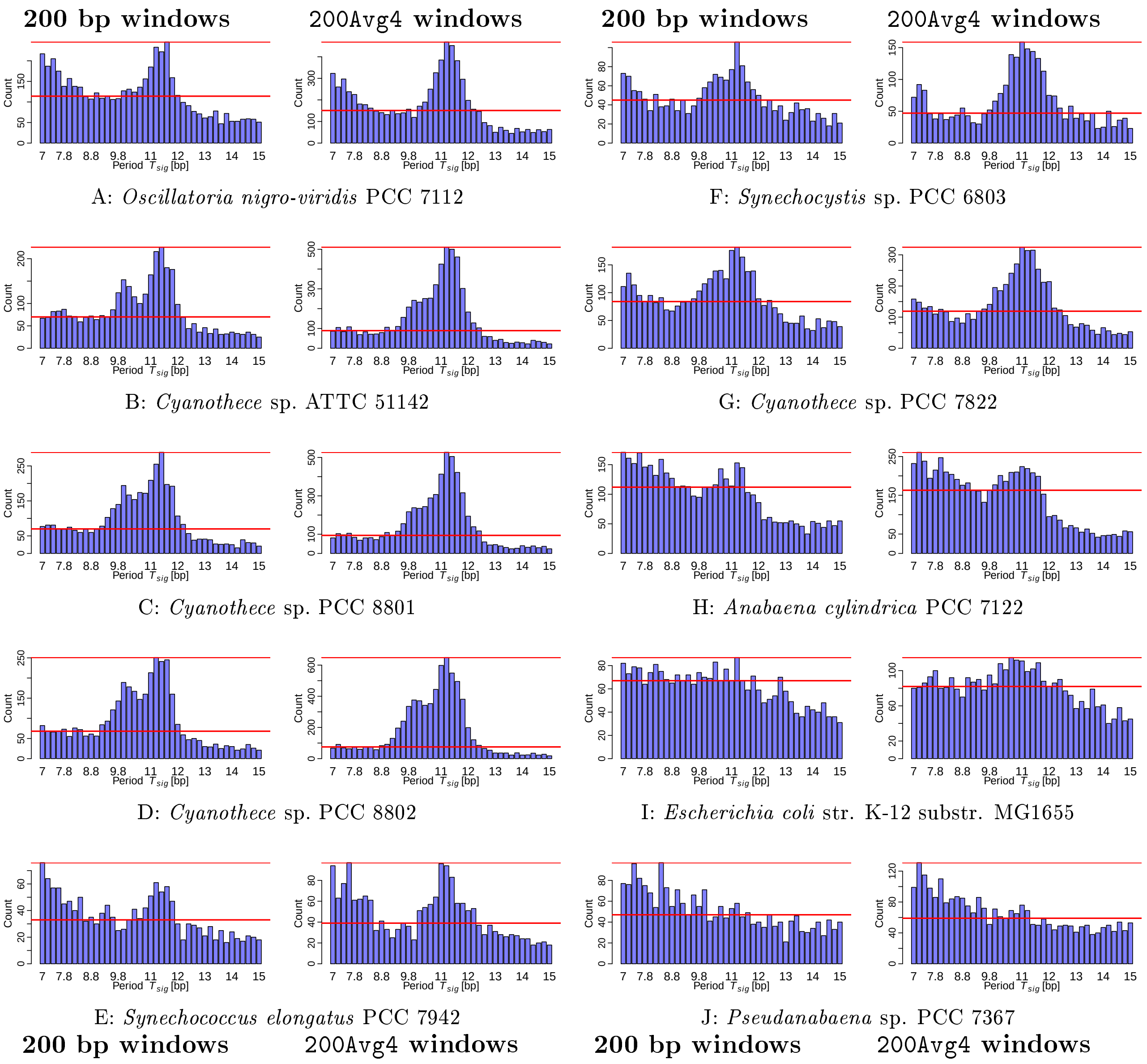

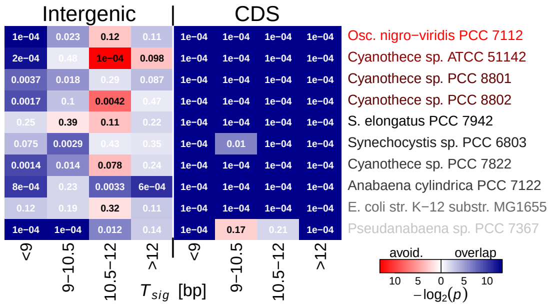

K: overlap tests for `200Avg4` windows

Supporting Figure S8: **Periodic Windows & Segments.** S8A–S8J: Histograms of the dominant periods T_{sig} of all genomic windows i with $\min(P_{T,i}) < 0.01$ for primary windows of 200 bp length (left plot in each panel) and with averaging over 4 adjacent spectra (`200Avg4`, right plot). Red lines indicate the maxima and medians of the T_{sig} distribution. S8K: same as Figure 3A of the main article but for the `200Avg4` windows. **Summary:** while Welch's method (`200Avg4`) yields smoother spectra and lower relative background signals (maximal *vs.* median counts, red lines), and a higher counts of significantly periodic windows at the expected periods, it decreases the resolution between bimodal peaks at ∼10 bp and ∼11 bp in *Cyanothece* sp. and *Synechocystis* sp. PCC 6803. The overlap test shows that the higher window size in `200Avg4` (800 bp) may penalize smaller isolated windows of high periodicity such as expected from intergenic regions (*cf.* promoter curvature). However, these larger significantly periodic windows (effective size 800 bp) are clearly associated with coding regions even in weakly periodic species.

3.2 CDS Periodicity Cluster in PCC 6803 und PCC 8801

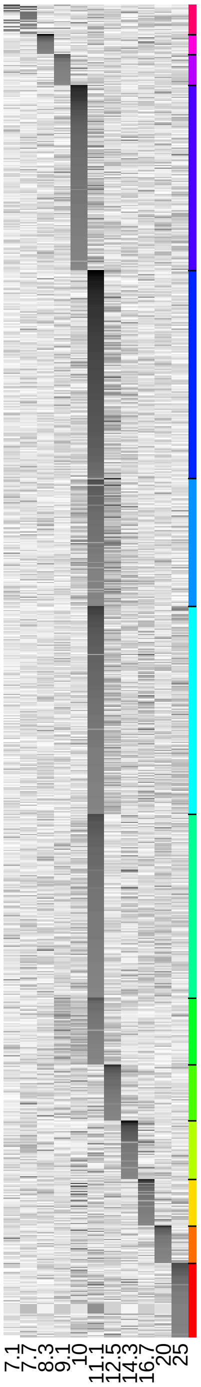

Supporting Figure S9: **Clustering of CDS AT2 periodicity spectra in *Cyanothece* sp. PCC 8801.** As Figure 5A of the main article but for the 1000 most periodic CDS of *Cyanothece* sp. PCC 8801. See Figure S10A for CDS counts and T_{max}.

	Synechocystis sp. PCC 6803						
**cluster**	**1**	**2**	**3**	**4**	**5**	**6**	**7**
T_{max}	7.1	7.7	8.3	9.1	10	11.1	11.1
count	27	26	30	34	73	202	23
group	"<=10"					"11.1"	
**cluster**	**8**	**9**	**10**	**11**	**12**	**13**	**14**
T_{max}	12.5	12.5	14.3	16.7	20	25	25
count	103	12	131	122	92	90	35
group	"12.5"		">14"				

	Cyanothece sp. PCC 8801						
**cluster**	**1**	**2**	**3**	**4**	**5**	**6**	**7**
T_{max}	7.7	8.3	9.1	10	11.1	11.1	11.1
count	22	15	23	139	156	96	156
**cluster**	**8**	**9**	**10**	**11**	**12**	**13**	**14**
T_{max}	11.1	11.1	12.5	14.3	16.7	20	25
count	138	50	42	44	35	28	56

A: CDS Cluster Counts and Periods

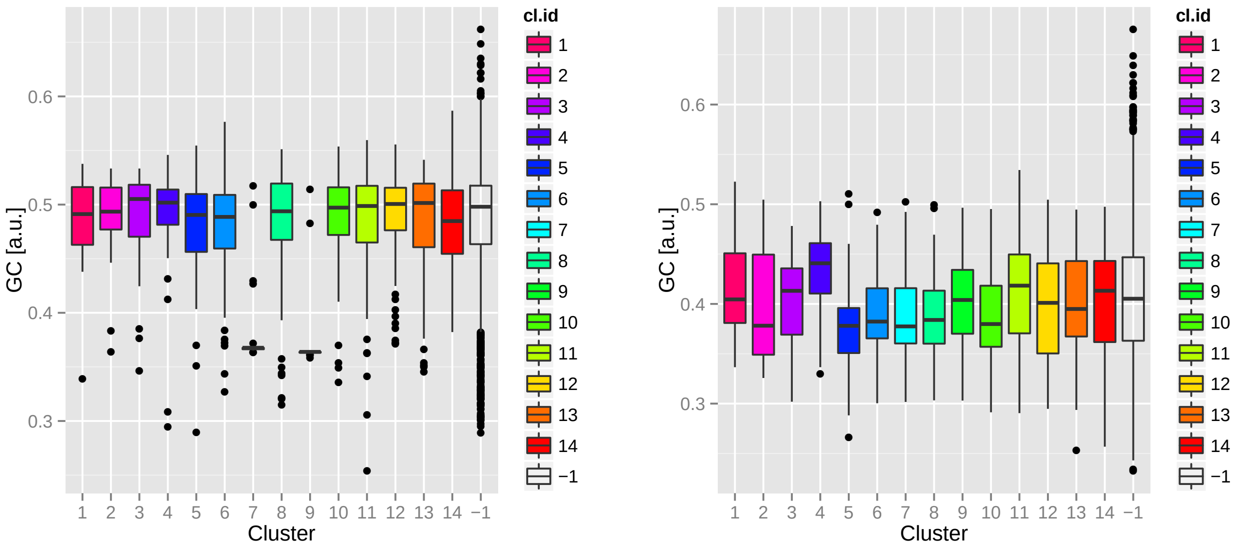

B: CDS Cluster GC Content

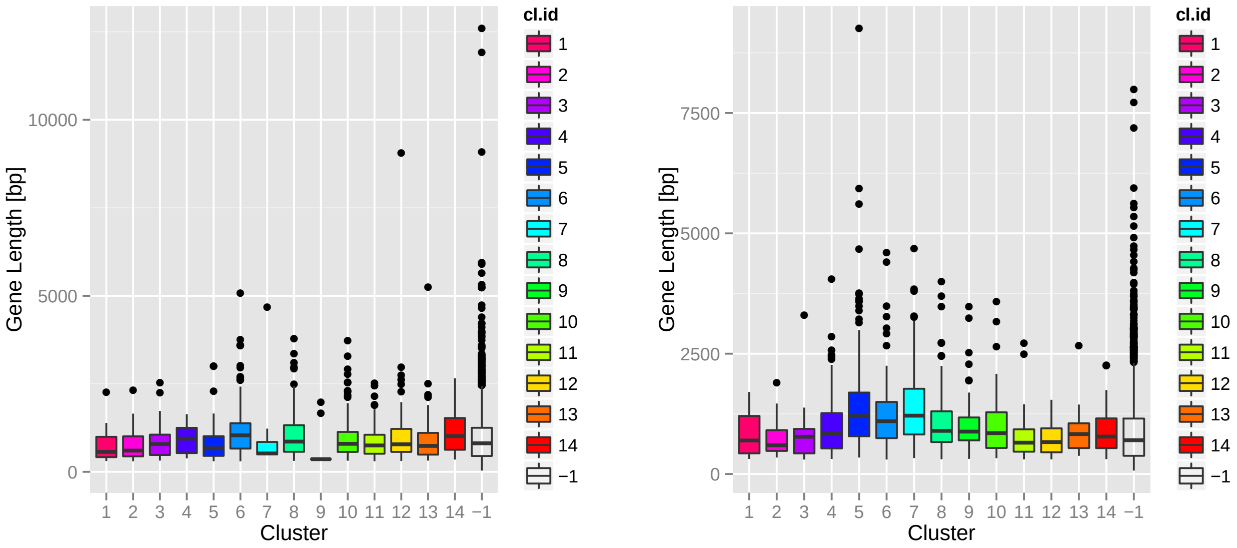

C: CDS Cluster Length

Supporting Figure S10: **Properties of CDS peridiocity clusters** in *Synechocystis* sp. PCC 6803 (left) and *Cyanothece* sp. PCC 8801 (right) CDS. S10A: The main periods and CDS counts (both) and the grouping of the CDS clusters (only *Synechocystis* sp. PCC 6803) shown in Figure 4A of the main manuscript and S9. S10B & S10C: Distributions of GC-content and gene lengths for all CDS clusters. The uncolored box contains all genes excluded from the spectral clustering (cluster "-1"). **Summary:** in PCC 8801 clusters 5–8, all with $T_{max} = 11.1$ bp tend to have a higher AT content and longer genes. Genes in the small clusters 7 and 9 in *Synechocystis* sp. PCC 6803 (T_{max} 11.1 and 12.5 bp) have an exceptionally high AT content. Cluster 7 are full length copies and 3' halves, and cluster 9 are 5' halves of the ISY100 transposon.

Cluster-wise Domain Annotation Enrichment 6803			
Domain-ID	p-value/BH	in Cluster	total
**Cluster 3 (30 genes, T_{max} 8.3 bp)**			
PF01850	0.0074/1	2	4
PF00395	0.0038/1	2	3
**Cluster 4 (34 genes, T_{max} 9.1 bp)**			
PF01609	0.0048/1	2	3
**Cluster 6 (202 genes, T_{max} 11.1 bp)**			
PF04055	0.0041/1	5	6
**Cluster 7 (23 genes, T_{max} 11.1 bp)**			
PF01710	3.1e-07/1.6e-4	7	20
**Cluster 9 (12 genes, T_{max} 12.5 bp)**			
PF01710	3.2e-16/1.7e-13	10	20
**Cluster 10 (131 genes, T_{max} 14.3 bp)**			
PF00805	9e-05/0.048	6	7
**Cluster 14 (35 genes, T_{max} 25 bp)**			
PF00070	0.01/1	2	4

Cluster-wise Domain Annotation Enrichment 8801			
Domain-ID	p-value/BH	in Cluster	total
**Cluster 1 (22 genes, T_{max} 7.7 bp)**			
PF01385	1.1e-4/0.054	4	11
**Cluster 3 (23 genes, T_{max} 9.1 bp)**			
PF01590	0.0072/1	2	5
**Cluster 7 (156 genes, T_{max} 11.1 bp)**			
PF00924	0.0068/1	3	3
**Cluster 8 (138 genes, T_{max} 11.1 bp)**			
PF01527	7.7e-4/0.37	4	4
**Cluster 11 (44 genes, T_{max} 14.3 bp)**			
PF00805	4.8e-14/2.3e-11	12	15
**Cluster 14 (56 genes, T_{max} 25 bp)**			
PF01609	3.6e-08/1.7e-05	7	8

Pfam Domain Index:

ID	Family	Description	T_{max}
PF01385	OrfB_IS605	Probable transposase	7.7 bp
PF01850	PIN	PIN domain	8.3 bp
PF00395	SLH	S-layer homology domain	8.3 bp
PF01590	GAF	GAF domain	9.1 bp
PF04055	Radical_SAM	Radical SAM superfamily	11.1 bp
PF00924	MS_channel	Mechanosensitive ion channel	11.1 bp
PF01527	HTH_Tnp_1	helix-turn-helix motif, transposase	11.1 bp
PF01710	HTH_Tnp_IS630	helix-turn-helix motif, transposase	11.1 & 12.5 bp
PF00805	Pentapeptide	Pentapeptide repeat	14.3 bp
PF00070	Pyr_redox	Pyridine nucleotide-disulphide oxidoreductase	14.3 bp
PF01609	DDE_Tnp_1	Transposase DDE domain	9.1 & 25 bp

Supporting Table S1: **Protein domain enrichments in CDS clusters.** Protein domain annotation enrichment of *Synechocystis* sp. PCC 6803 (left) and *Cyanothece* sp. PCC 8801 (right) CDS periodicity clusters in Figure 4A of the main article and Figure S9. The p-values were calculated by cumulative hypergeometric distribution tests and p-values before ("p-value") and after adjustment for multiple testing by the Benjamini-Hochberg method ("BH") are shown. Individual domains from the prediction algorithms HMMPfam, HMMTigr, Gene3D, HMMPanther, HMMSmart, BlastProDom, ProfileScan, FPrintScan, and HMMPIR were tested for PCC 6803 (total: 6813 domains) and PCC 8801 (total: 6599 domains). All overlaps that were significant ($p \leq 0.1$) before BH control are shown. For both species only HMMPfam annotations yielded significant results. The domain names and descriptions of enriched domains are provided together with the main periods T_{max} of the respective CDS periodicity clusters. **Summary:** the pentapeptide repeat domain (PF00805) which coincidentally may mimic DNA structure and inhibit DNA gyrase [97], is found in clusters with $T_{max} = 14.3$ bp in both species and likely reflects secondary effects of the amino acid code (pentapeptide repeat: 5 amino acids = 15 bp). None of the enrichments could comprehensively explain the CDS periodicity profiles by such secondary effects.

Cluster-wise Category Annotation Enrichment 6803			
Category	p-value/BH	in Cluster	total
**Cluster 2 (26 genes, T_{max} 7.7 bp)**			
Chaperones	0.007/0.48	2	16
**Cluster 5 (73 genes, T_{max} 10 bp)**			
Fatty acid, phospholipid and sterol metabolism	0.0076/0.14	3	39
**Cluster 7 (23 genes, T_{max} 11.1 bp)**			
Other categories	2.6e-18/4.6e-17	20	305
Transposon-related functions	1.6e-23/1.1e-21	18	107
**Cluster 9 (12 genes, T_{max} 12.5 bp)**			
Other categories	5.1e-11/9.2e-10	11	305
Transposon-related functions	6.7e-14/4.6e-12	10	107
**Cluster 13 (90 genes, T_{max} 25 bp)**			
Soluble electron carriers	6.1e-4/0.042	4	15
**Cluster 14 (35 genes, T_{max} 25 bp)**			
Cellular processes	0.0086/0.15	4	76
NADH dehydrogenase	0.0016/0.11	3	22

Cluster-wise Category Annotation Enrichment 8801			
Category	p-value/BH	in Cluster	total
**Cluster 1 (22 genes, T_{max} 7.7 bp)**			
Replication, recombination and repair	2.8e-4/0.0066	5	120
**Cluster 4 (139 genes, T_{max} 10 bp)**			
Amino acid transport and metabolism	0.0056/0.079	11	142
Translation, ribosomal structure and biogenesis	0.0066/0.079	11	145
**Cluster 5 (156 genes, T_{max} 11.1 bp)**			
Replication, recombination and repair	0.0038/0.077	11	120
Coenzyme transport and metabolism	0.0064/0.077	10	111
**Cluster 7 (156 genes, T_{max} 11.1 bp)**			
Secondary metabolites biosynthesis, transport and catabolism	0.0071/0.085	6	48
Defense mechanisms	0.0036/0.085	6	42
**Cluster 8 (138 genes, T_{max} 11.1 bp)**			
Multiple Categories	0.009/0.22	17	286
**Cluster 11 (44 genes, T_{max} 14.3 bp)**			
Function unknown	5.4e-07/1.3e-05	15	338

Supporting Table S2: **Function enrichments in CDS clusters.** As Table S1 but for CyanoBase Function categories of *Synechocystis* sp. PCC 6803 (top, total: 84 categories) and *Cyanothece* sp. PCC 8801 (bottom, total: 23 categories).

3.3 Supercoiling-sensitive and Diurnal Transcription in PCC 6803

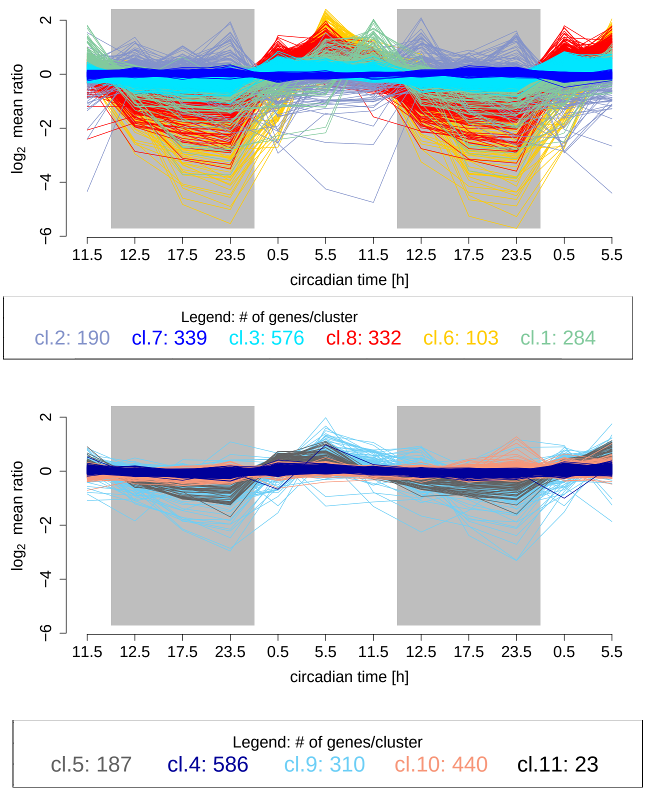

A: Diurnal transcriptome timecourses

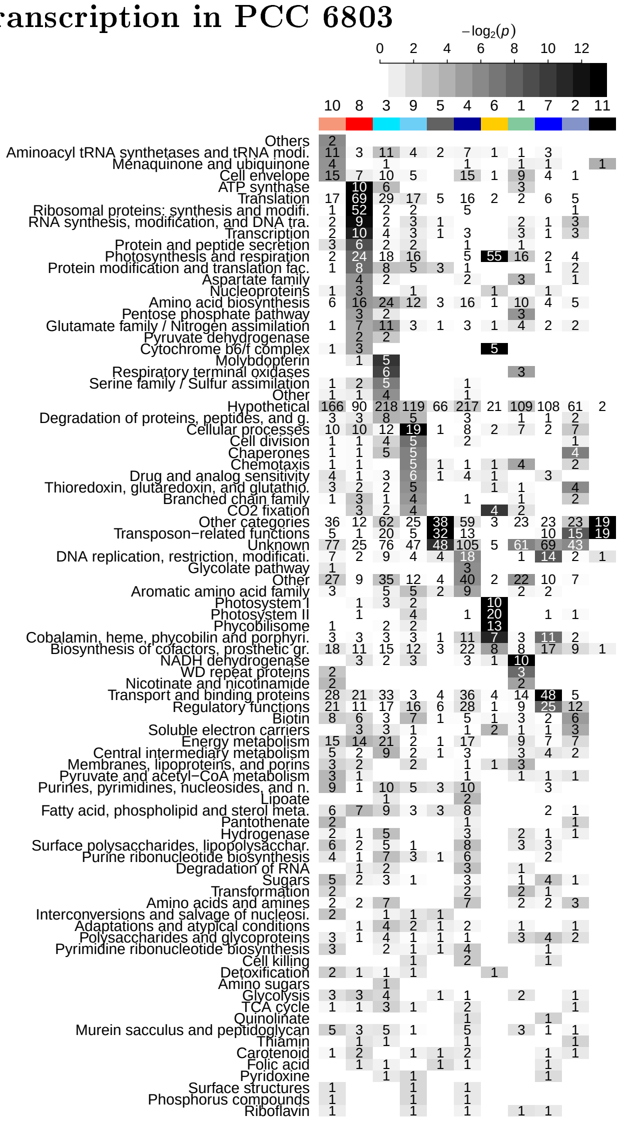

B: Diurnal transcriptome annotation

Supporting Figure S11: **Diurnal co-transcription cohorts in *Synechocystis* sp. PCC 6803**, data from ref. [46]. S11A: micro-array-based time-series for 3370 protein-coding transcripts; the Discrete Fourier Transforms of timeseries (concatenation of two biological duplicates) were clustered into co-transcribed cohorts (color-code) by the model-based clustering tool `flowClust` [48] as previously described [46,47], and choosing cluster number $k = 10$. Cohort 11 comprises 23 annotated genes that were not probed on the microarray. For this visualization only (but not for clustering) the data was normalized by the 'least-oscillating set' of transcripts [46,47]. Dark-phases are indicated by gray boxes. The log2 of the mean ratio is shown. Cohorts were sorted by phase and split into two groups for clarity of the plot. S11B: overlap of co-transcribed cohorts with functional annotations (obtained from CyanoBase), where significance was tested by cumulative hypergeometric distribution tests without control for multiple testing to show an unbiased functional profile.

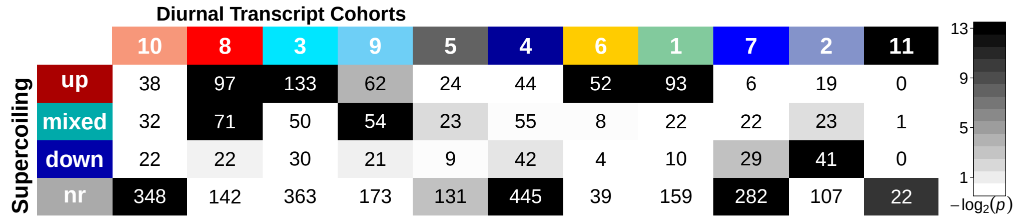

A: Diurnal vs. Supercoiling-sensitive transcripts

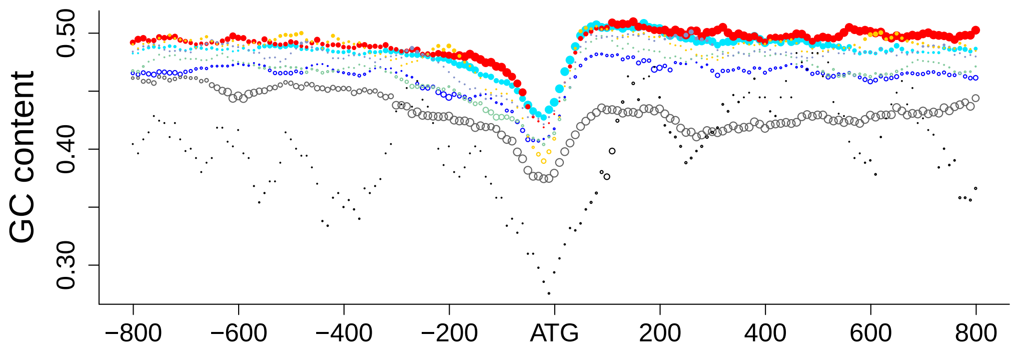

B: GC content of supercoiling-sensitive genes

Supporting Figure S12: **Diurnal vs. supercoiling-sensitive transcription in *Synechocystis* sp. PCC 6803.** S12A: overlap (numbers) and p-values from cumulative hypergeometric distribution tests (color-code, see legend) of the diurnal co-expression cohorts (Fig. S11) with transcript groups defined as supercoiling-sensitive (up: activated by negative supercoiling in a series of experiments, down: deactivated, mixed: mixed response, nr: non-responsive) in [49]. S12B: the GC-content (in moving windows of 71 bp) of supercoiling-sensitive diurnal cohorts around the start codon (ATG) is shown as statistical DNA profiles (SDP, described in ref. [47]), where the size of the circles inversely correlates with the p-value ($\sim -\log_2(p)$) of two-sided t-tests (cohort vs. total distribution in all other annotated protein-coding genes) at each x coordinate; filled and open circles indicate values higher or lower then the total genome average. Note, that genes in periodicity clusters 7 & 9 were removed from diurnal cohort 5 (gray circles) and not-on-array cohort 11 (black circles and dots) to show that AT-richness of these cohorts persists. **Summary:** The diurnal cohorts show the expected dependency on DNA supercoiling and the typical bias in GC/AT-content of supercoiling-sensitive transcription (see main manuscript), as previously observed in diurnal and gyrase-dependent transcription in *Synechococcus elongatus* PCC 7942 [35] and supercoiling-sensitive transcription in *E. coli* [62] [98].

3.4 Transposons in PCC 6803 und PCC 8801

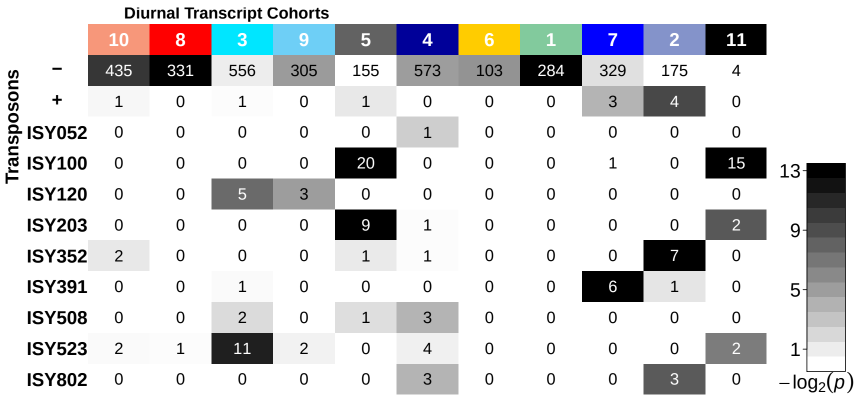

A: Transposons and Diurnal Transcriptome Cohorts

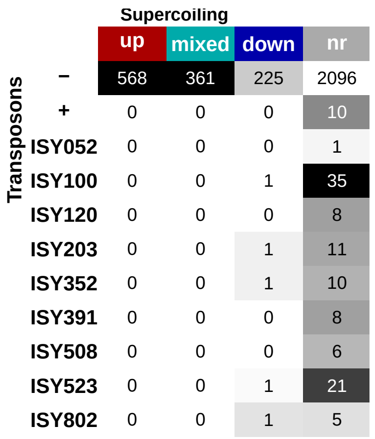

B: Transposons and Supercoiling-sensitive Transcription

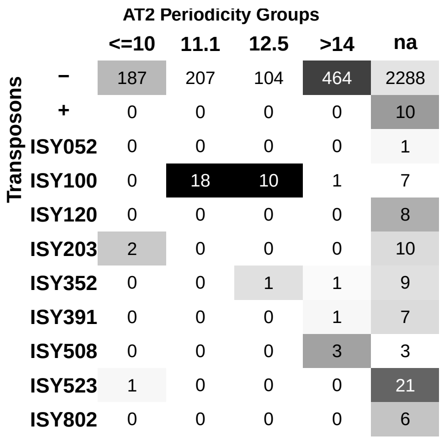

C: Transposons and CDS AT2 Periodicity Groups

Supporting Figure S13: **Transposons in *Synechocystis* sp. PCC 6803.** Transposons annotated in *Synechocystis* sp. PCC 6803: ISYxxx are named transposons (multiple copies), + indicates not further specified transposon genes and - are all other annotated CDS. The figures show their overlap profiles with the diurnal transcriptome cohorts (S13A) from Figures 4C & S11, with supercoiling-sensitive transcript groups (S13B) from ref. [49] & Figure S12, and with CDS AT2 periodicity groups (S13C) from Figures 4A and S10A, S1 & S2. Note, that many transposons are annotated as "partial copies", where annotated 5' and 3' halves are direct neighbors on the genome. **Summary:** ISY100 is the most abundant transposon in the genome of *Synechocystis* sp. PCC 6803. Note that some of the annotations are actually separately annotated but adjacent 5' and 3' halves; 22 copies have been reported in a detailed analysis [68]. Other transposons are also significantly associated with certain transcriptome cohorts. However, in a given transposons are all paralogous copies and microarray may not properly distinguish between individual copies. None of the transposons showed explicit dependency on supercoiling and only ISY100 showed a specific AT2 periodicity profile. ISY100 is co-expressed in diurnal cohort 5 with other AT-rich genes of unknown function but in-phase with GC-rich and supercoiling-activated growth genes (Fig. S11 & S12).

```
                                                                            M  A  Y  S  L
TATAGTCATTTCAATTAACGATGAGAGAATTTAATGTAAAATTATGGAGTGTACAAAATGAACAGGTTTAAACAATGGCTTACAGTTTAG
         10        20        30        40        50        60        70        80        90

D  L  R  Q  R  V  V  A  Y  I  E  A  G  G  K  I  T  E  A  S  K  I  Y  K  I  G  K  A  S  I
ATTTAAGGCAAAGGGTAGTAGCTTATATAGAAGCTGGAGGAAAAATAACTGAGGCTTCCAAGATATATAAAATAGGAAAAGCCTCGATAT
        100       110       120       130       140       150       160       170       180

Y  R  W  L  N  R  V  D  L  S  P  I  K  V  E  R  R  H  R  K  L  D  W  E  A  L  K  K  D  V
ACAGATGGTTAAATAGAGTAGATTTAAGCCCAATAAAAGTAGAGCGTCGCCATAGGAAATTAGACTGGGAAGCTCTAAAAAAAGACGTAG
        190       200       210       220       230       240       250       260       270

E  E  N  P  D  A  R  L  I  D  R  A  K  K  F  G  V  R  P  S  A  V  Y  Y  A  L  K  K  M  K
AAGAAAATCCCGATGCAAGATTGATAGACAGAGCCAAGAAATTTGGAGTGAGGCCGAGTGCCGTATATTACGCATTAAAGAAAATGAAAA
        280       290       300       310       320       330       340       350       360

I  N  R  K  K  K  E  L  R  Y  R  E  R  N  R  E  E  R  V  K  Y  Y  R  M  L  R  E  L  I  K
TAAACAGAAAAAAAAAAGAACTACGTTATCGAGAAAGAAACCGGGAGGAACGAGTTAAGTACTATAGAATGTTAAGAGAACTAATTAAGC
        370       380       390       400       410       420       430       440       450

L  Y  G  S  Q  A  I  V  Y  I  D  E  S  G  F  E  A  I  Q  A  C  I  Y  A  W  S  K  K  G  K
TCTATGGTAGTCAAGCTATAGTTTACATAGATGAATCTGGATTCGAAGCAATCCAGGCTTGTATTTATGCCTGGTCAAAAAAAGGAAAAA
        460       470       480       490       500       510       520       530       540

K  V  Y  G  D  R  Q  G  K  R  G  V  R  E  N  L  V  A  G  R  R  K  G  K  K  D  L  I  A  P
AAGTTTATGGAGATAGACAAGGAAAAAGGGGAGTCAGAGAAAATCTAGTAGCAGGGAGAAGAAAAGGAAAAAAAGATTTGATTGCGCCGA
        550       560       570       580       590       600       610       620       630

M  V  F  T  G  S  L  N  A  E  G  F  E  G  W  L  K  L  Y  L  L  P  S  L  D  I  P  S  I  L
TGGTTTTTACCGGGAGTTTGAATGCAGAAGGCTTTGAAGGATGGTTAAAATTATATTTGCTACCCTCCCTCGACATTCCATCAATATTAA
        640       650       660       670       680       690       700       710       720

I  M  D  N  A  P  I  H  R  K  T  A  I  K  E  L  A  K  E  A  G  H  E  V  L  F  L  P  K  Y
TAATGGATAATGCTCCTATTCATCGTAAAACTGCCATTAAAGAATTGGCTAAAGAAGCAGGTCATGAAGTTCTTTTTTTGCCGAAATATT
        730       740       750       760       770       780       790       800       810

S  P  D  L  N  D  I  E  H  D  F  S  A  L  K  R  A  R  M  Y  A  P  I  D  T  S  L  D  E  I
CTCCTGATTTAAATGATATTGAGCATGACTTTAGTGCCTTGAAACGAGCTAGAATGTACGCTCCTATTGACACGTCTCTTGATGAAATTA
        820       830       840       850       860       870       880       890       900

I  R  S  Y  C  G  V  .
TCCGTTCTTACTGTGGCGTTTAGCGTCTCAGCTTTATTTGAAACTACTATA
        910       920       930       940       950
```

Supporting Figure S14: **Sequence of the ISY100f transposon** of *Synechocystis* sp. PCC 6803. Sequences features are colored as in Figure 5 (top) of the main article and according to the transposon primary structure as reported in ref. [68]: left and right inverted repeats (red), the PFAM domain PF0170 (blue) and the 3' half of the transposase ORF. The transposase amino acid sequence is indicated (centered on codons).

```
M P S N P Q L N L M T N L L Q L E G V T V I N Y Q I I K E I
ATGCCATCAAATCCTCAACTAAATTTAATGACTAACCTGTTACAACTAGAAGGAGTGACAGTCATCAATTATCAGATAATAAAAGAGATA
10 20 30 40 50 60 70 80 90

G I V L S V E K I E P N A T C I Y C G S K T R K V H Q N N E
GGAATAGTTTTATCTGTAGAGAAAATAGAGCCAAATGCTACCTGTATTTACTGTGGTTCAAAAACGAGGAAAGTTCATCAAAATAACGAA
100 110 120 130 140 150 160 170 180

L T I R D L P W G E K S V Y L K I N R R Q M R C E H C Q K K
TTAACAATTAGGGATTTACCCTGGGGAGAAAAATCGGTTTATTTAAAAATTAATCGTCGGCAAATGAGATGTGAGCATTGTCAAAAGAAA
190 200 210 220 230 240 250 260 270

F T E E L S Y L P K K R T Y T E R F R K K I I E E V L N S D
TTCACAGAGGAATTGAGTTATTTGCCCAAAAAAAGAACTTATACTGAGAGATTTAGAAAGAAAATAATTGAAGAAGTTTTAAATAGTGAC
280 290 300 310 320 330 340 350 360

I K N V A K R N G V S E Q E I E T M L K D V G E D L N Q E K
ATCAAGAATGTAGCGAAAAGAAATGGAGTTAGTGAACAAGAAATAGAAACGATGCTGAAAGATGTAGGAGAAGACTTAAACCAAGAAAAA
370 380 390 400 410 420 430 440 450

P R E L R R L G I D E I A V I K G Q G N Y Y V V L V D L E R
CCGAGGGAATTAAGGCGATT AGGAATTGATGAAATCGCGGTGATTAAAGGACAAGGAAATTATTATGTTGTCTTAGTTGATTTAGAGAGA
460 470 480 490 500 510 520 530 540

G V I V G I L E K R I E E E V L K Y L E A W G E E V L T K I
GGAGTGATAGTAGGAATTCTAGAAAAACGAATAGAAGAGGAAGTTTTAAAATATCTAGAAGCATGGGGAGAAGAGGTTTTGACGAAGATT
550 560 570 580 590 600 610 620 630

K E V S I D L W K P Y K N I V N K L M P Q A E V V A D R F H
AAAGAAGTGAGTATAGATCTTTGGAAACCTTATAAAAATATTGTGAATAAATTAATGCCCCAAGCTGAAGTCGTAGCTGATAGATTTCAT
640 650 660 670 680 690 700 710 720

V M K Q V N E E L D A Q R K T L K R E A K E L K D T N Q K E
GTAATGAAACAAGTTAATGAGGAATTAGATGCTCAAAGAAAAACT CTTAAAAGAGAAGCTAAAGAGCTAAAAGATACTAATCAAAAAGAA
730 740 750 760 770 780 790 800 810

E I L S G L N K S K Y V L L K N E E D L N E E Q K E K L E Q
GAAATATTGTCAGGATTAAATAAGAGTAAATATGTTTTATTGAAAAATGAAGAAGATTTAAACGAAGAGCAAAAAGAAAAATTAGAGCAA
820 830 840 850 860 870 880 890 900

V Y K T S E V L S K M H Q L K E E F R D I F E T Q S D W V S
GTCTATAAAACGTCAGAAGTCCTATCAAAAATGCACCAATTGAAGGAGGAATTTAGAGACATTTTTGAAACCCAGTCTGACTGGGTTTCA
910 920 930 940 950 960 970 980 990

G L F E L A N W C Q K A Y S L Y P K S C G T I R R W I G E I
GGACTATTTGAATTAGCAAATTGGTGTCAAAAGGCTTATTCATTGTACCCGAAAAGTTGTGGAACAATTAGGCGTTGGATTGGAGAAATT
1000 1010 1020 1030 1040 1050 1060 1070 1080

I A Y F D Q G T T Q G I V E G I N N K L K L I K R R A Y G F
ATTGCCTATTTTGACCAAGGAACAACTCAAGGAATAGTCGAAGGTATTAACAATAAATTAAAGTTGATTAAAAGGAGAGCTTATGGCTTT
1090 1100 1110 1120 1130 1140 1150 1160 1170

R N F G N F Q L R S F L T W H F T R .
AGAAATTTTGGTAATTTTCAACTCAGAAGTTTCTTAACTTGGCATTTTACTCGTTAA
1180 1190 1200 1210 1220
```

Supporting Figure S15: **Sequence of the PCC8801_2977 transposase ORF** of *Cyanothece* sp. PCC 8801. Sequences features are colored as in Figure 5 (bottom) of the main article: the annotated PFAM domain (PF01610, in blue) and rest of the ORF in yellow. The transposase amino acid sequence is indicated (centered on codons).

References

[94] Grosse, I., Herzel, H., Buldyrev, S., and Stanley, H. (2000) Species independence of mutual information in coding and noncoding DNA.. *Phys Rev E Stat Phys Plasmas Fluids Relat Interdiscip Topics,* **61**(5 Pt B), 5624–5629.

[95] Paradis, E., Claude, J., and Strimmer, K. (2004) APE: Analyses of Phylogenetics and Evolution in R language.. *Bioinformatics,* **20**(2), 289–290.

[96] Kampstra, P. (2008) Beanplot: A Boxplot Alternative for Visual Comparison of Distributions. *Journal of Statistical Software, Code Snippets,* **28**(1), 1–9.

[97] Hegde, S., Vetting, M., Roderick, S., Mitchenall, L., Maxwell, A., Takiff, H., and Blanchard, J. (2005) A fluoroquinolone resistance protein from *Mycobacterium tuberculosis* that mimics DNA.. *Science,* **308**(5727), 1480–1483.

[98] Blot, N., Mavathur, R., Geertz, M., Travers, A., and Muskhelishvili, G. (2006) Homeostatic regulation of supercoiling sensitivity coordinates transcription of the bacterial genome.. *EMBO Rep,* **7**(7), 710–715.
